# Non-Phenomenological Description of the Time-Resolved Emission in Solution with Quantum–Classical Vibronic Approaches—Application to Coumarin C153 in Methanol

**DOI:** 10.3390/molecules28093910

**Published:** 2023-05-05

**Authors:** Javier Cerezo, Sheng Gao, Nicola Armaroli, Francesca Ingrosso, Giacomo Prampolini, Fabrizio Santoro, Barbara Ventura, Mariachiara Pastore

**Affiliations:** 1 Departamento de Química and Institute for Advanced Research in Chemical Sciences (IAdChem), Universidad Autónoma de Madrid, 28049 Madrid, Spain; javier.cerezo@uam.es; 2 Institute of Chemistry of OrganoMetallic Compounds (ICCOM), National Research Council of Italy (CNR), Area di Ricerca di Pisa, Via Moruzzi 1, I-56124 Pisa, Italy; 3 Institute for Organic Synthesis and Photoreactivity (ISOF), National Research Council of Italy (CNR), Via P. Gobetti 101, I-40129 Bologna, Italy; 4 Université de Lorraine & CNRS, Laboratoire de Physique et Chimie Théoriques (LPCT), F-54000 Nancy, France

**Keywords:** time-resolved emission spectroscopy, mixed quantum chemical approaches, force field parameterization, molecular dynamics, time-dependent density functional theory, vibronic approaches, organic dyes

## Abstract

We report a joint experimental and theoretical work on the steady-state spectroscopy and time-resolved emission of the coumarin C153 dye in methanol. The lowest energy excited state of this molecule is characterized by an intramolecular charge transfer thus leading to remarkable shifts of the time-resolved emission spectra, dictated by the methanol reorganization dynamics. We selected this system as a prototypical test case for the first application of a novel computational protocol aimed at the prediction of transient emission spectral shapes, including both vibronic and solvent effects, without applying any phenomenological broadening. It combines a recently developed quantum–classical approach, the adiabatic molecular dynamics generalized vertical Hessian method (Ad-MD|gVH), with nonequilibrium molecular dynamics simulations. For the steady-state spectra we show that the Ad-MD|gVH approach is able to reproduce quite accurately the spectral shapes and the Stokes shift, while a ∼0.15 eV error is found on the prediction of the solvent shift going from gas phase to methanol. The spectral shape of the time-resolved emission signals is, overall, well reproduced, although the simulated spectra are slightly too broad and asymmetric at low energies with respect to experiments. As far as the spectral shift is concerned, the calculated spectra from 4 ps to 100 ps are in excellent agreement with experiments, correctly predicting the end of the solvent reorganization after about 20 ps. On the other hand, before 4 ps solvent dynamics is predicted to be too fast in the simulations and, in the sub-ps timescale, the uncertainty due to the experimental time resolution (300 fs) makes the comparison less straightforward. Finally, analysis of the reorganization of the first solvation shell surrounding the excited solute, based on atomic radial distribution functions and orientational correlations, indicates a fast solvent response (≈100 fs) characterized by the strengthening of the carbonyl–methanol hydrogen bond interactions, followed by the solvent reorientation, occurring on the ps timescale, to maximize local dipolar interactions.

## 1. Introduction

Understanding and predicting optical properties of molecular systems in condensed phase and complex environments is pivotal in different research areas, spanning from environmental science, biochemistry and optoelectronics to renewable energy harvesting and conversion [1,2,3,4,5,6,7,8,9,10]. The accurate simulation of UV–Vis light absorption and emission spectra has indeed been proven to be a precious tool in the conception of optimized molecular-based photoresponsive materials [11,12,13,14,15,16,17,18,19,20,21,22]. For steady-state spectroscopies, effective mixed quantum–classical (MQC) strategies have recently been proposed [23,24,25,26,27,28,29,30,31,32,33,34,35] and are nowadays mature enough to allow for a non-phenomenological description of the role of all the microscopic factors that contribute to the spectral shapes, delivering predictions which compare nicely with experiments. In particular these approaches are able to simultaneously account for the effect of the environment, usually described with classical molecular dynamics (MD), and of the quantum nature of the molecular vibrations, which are responsible for the underlying pattern of vibronic transitions, playing a major role in both well-resolved and structureless spectra [26,36]. Notwithstanding the power of steady-state measurements, a more in-depth comprehension of the photo-responsive and photo-reactive molecular properties, and of the role played by the environment, requires time-resolved (TR) experiments. Among TR techniques, pump–probe spectroscopy is definitely the most widely employed approach to study ultrafast excited state dynamics [37]. In a pump–probe set up, a fraction of the molecules is excited (pump pulse) to a resonant electronic state and, after a certain time delay (τ), a probe pulse is used to irradiate the sample and monitor the pump-induced changes in absorption. Pump–probe spectra show distinct, but often overlapping, features coming from the ground-state bleaching (GSB), stimulated emission (SE), and excited-state absorption (ESA) contributions, thus making the spectra appearance quite intricate and its interpretation far from being straightforward. Therefore the ability to reliably simulate excited state dynamics and excited state absorption/emission spectra through calculations would greatly support interpretation and assignment of experimental results [38,39].

Unfortunately, in most of the systems of practical interest, the simulation of TR spectra is much more challenging than for steady-state signals, due to one or more of the following factors: (i) the large number of excited states that one needs to take into account; (ii) the accurate treatment of higher-energy excited states, often dominated by double and higher-order excitations; (iii) the possibility of non-adiabatic interactions and photophysical/photochemical events; (iv) the necessity to account for the dynamical response of the environment to the electronic transition of the molecular probe. Although the theoretical foundations for these simulations are assessed and described in seminal works [40,41], we do not yet have computational protocols of general and straightforward applicability to predict TR UV-Vis spectra. Nonetheless, many approaches have been recently introduced, mostly focusing on different aspects of the general phenomenon, with varied computational burden [38,39,42,43,44,45,46,47,48,49,50]. When a single long-living (nanoseconds and more) excited state is populated in the ultrafast regime or TR (stimulated) emission is the target, simulations of transient signals is simpler as, usually, the calculations may focus on only one state [51,52,53,54]. This is the case, for instance, of transition metal complexes such as ruthenium polypyridyl compounds, which have been largely exploited in the study of photoinduced charge-transfer processes [55,56], as well as of the luminescent organic molecular probes [57]. Even in this simplified scenario, as happens for steady-state signals, the spectral lineshape generally arises from the interplay of both vibronic and environmental effects, since they both have an impact on energies, transition amplitudes and excited state relaxation pathways [30,35,44,58,59,60,61,62,63]. The scope of this contribution is, therefore, to propose a possible route to extend the MQC methodologies developed for steady-state spectroscopies to TR emission of long-living excited states, analyzing its computational effectiveness and performance.

In such a framework, continuum solvent models [64], although computationally appealing, can only capture bulk polarization effects and predict with reasonable accuracy solvatochromic shifts not related to specific solvent–solute interactions. Moreover, in most implementations, they do not explicitly account for the time dependence of the solvent response. Hence they are not, in general, a viable route to track solvent reorganization effects following the electronic excitation. Two main ingredients are instead required to accomplish this task, i.e., accurate FFs mimicking both the ground and the excited state, and nonequilibrium MD simulations [52,65,66], resulting in a significant computational overhead. In fact, besides the computational burden arising from excited state FF parametrization, a nonequilibrium ensemble average requires a prior equilibrium MD run in the ground state, sufficiently long to extract a series (hundreds) of uncorrelated solute/solvent configurations, to initiate from each of them nonequilibrium runs in the excited state. Recently, some of us reported on the dynamic reorganization of the solvent and its impact on the transient UV-Vis spectral evolution of a prototypical Ru(II)-polypyrydil complex, by combining quantum mechanical (time-dependent density functional theory, TD-DFT) calculations with equilibrium and nonequilibrium classical MD simulations. The latter were performed employing accurate quantum mechanical derived force fields (QMD-FFs) [67,68], specifically parameterized for the ground and the excited state under consideration [51,52]. This multi-level approach was able to accurately reproduce the experimental spectral evolution and to associate the observed spectral shift to specific short- and long-range solvent reorganization effects. Despite these encouraging results achieved with QMD-FFs, it should be noted that the calculated spectral shapes are still undermined by the MD treatment of the nuclei, whose dynamics is sampled according to classical physics, thus preventing to correctly account for the manifold of vibrational transitions due to the quantum nature of nuclear vibrations.

In the present work we aim at filling this lack, by extending to TR emission the adiabatic-MD generalized vertical Hessian (Ad-MD|gVH) approach, a MQC method recently proposed by some of us [31], and until now applied, to our knowledge, only in the calculation of steady-state absorption and emission spectra [31,69]. The Ad-MD|gVH approach was originally designed to describe the shape of the electronic spectra of flexible systems in condensed phase and is based on a partition of the nuclear degrees of freedom (DoFs) of the solute+environment super-system in soft modes (treated classically) and stiff modes (treated quantum-mechanically). The two subsets of DoFs are coupled through an adiabatic approximation which considers quantum modes fast enough to rearrange instantaneously to any fluctuation of the soft modes. The latter are sampled with classical MD, carried out with accurate QMD-FFs. Here we apply the TR extension of the Ad-MD|gVH approach to a well known benchmark dye, namely coumarin 153 (C153), that was originally developed as one of the laser dyes used in the blue–green region and displays high quantum yields of fluorescence in a wide variety of polar solvents [70,71]. The first excited state (S${}_{1}$) of this molecule is relatively low-lying in energy and is characterized by a large change in the molecular dipole moment compared with the ground electronic state (S${}_{0}$), thus leading to a large Stokes shift. In addition, the S${}_{0}\to$ S${}_{1}$ excitation involves a partial charge transfer from the amino (electron donor) group to the carbonyl (electron accepting) group, stabilizing the excited state [72]. Regarding the steady state (absorption and emission) spectra of C153, a vibronic structure appears in nonpolar solvents, whereas it is not displayed when the spectra are recorded in polar solvents [73]. Due to the strong Stokes shift effect and the dipolar character of the S${}_{0}\to$ S${}_{1}$ excitation, C153 also represents one of the favorite probes of solvation dynamics [73]. The steady-state spectra of C153, and their change from gas-phase to polar (aprotic) solvents, have been successfully simulated and interpreted in the past, including by some of us [59,74], indicating that, even in polar solvents where the spectrum is structureless, its shape is determined by the combination of the effects of intramolecular vibrations and the solvent effects. Therefore, this system appears to be a naturally ideal test case for our new approach. In summary, here we present a combined experimental theoretical work on the steady-state absorption and emission, and on the TR emission of C153. As a medium we selected methanol, a protic solvent that can establish specific interactions with C153, sensitive to its electronic state, which cannot be described by continuum models adopted in the past [59]. Despite the fact that many experimental data have already been published in early studies on C153 [72,73,75,76,77,78,79,80], we here decided to retake the experimental characterization of the system, by measuring steady-state and TR spectra and determining the excited state lifetime, so as to have a complete and consistent set of data to compare with the predictions of the proposed computational approach.

## 2. Results and Discussion

### 2.1. Experimental Steady-State Absorption and Emission Spectra and Transient Absorption Analysis

The photophysical properties of C153 have been measured in methanol solution at room temperature and at 77 K.

**Figure 1:**
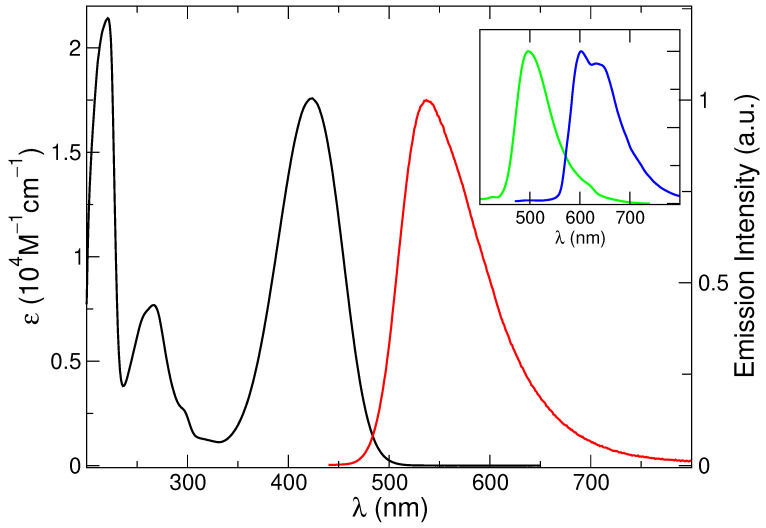
Absorption (black line: left axis) and emission spectrum (right axis, $\lambda_{exc}=380$ nm) of C153 in methanol at 298 K (red line). In the inset, the emission spectrum at 77 K (green line) and the phosphorescence spectrum in 1:1 methanol-C${}_{2}$H${}_{5}$I solution at 77 K (blue line) are also included.

The absorption spectrum, reported in Figure 1, shows three main bands at 221, 266 and 424 nm (Table 1), in agreement with literature reports [77]. The steady-state fluorescence spectrum appears as a broad asymmetric band peaking at 537 nm (Figure 1). An excited state lifetime of 4.0 ns and an emission quantum yield of 42% was measured (Table 1) [75,81].

Spectra recorded in glassy matrix at 77 K revealed a blue-shifted and narrower fluorescence profile (maximum at 497 nm) and the use of a heavy atom containing solvent (methanol/ethyl iodide 1:1 mixture) enabled the observation of a vibrationally resolved phosphorescence band (Figure 1 and Table 1). These results confirm the rich photophysics of this compound. In the present study, however, we will focus on the absorption and emission spectra at 298 K.

**Figure 2:**
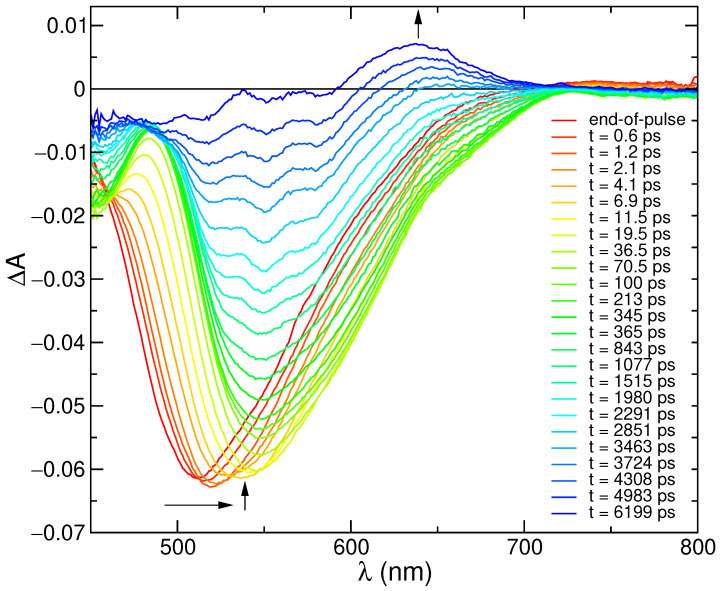
Transient absorption spectra of C153 in methanol at the end of pulse and at different delays. Excitation at 350 nm (A${}_{350}$ = 0.2, 0.2 cm optical path, 6 μJ/pulse).

In order to get insight into the photophysical processes occurring at early times, transitient absorption (TA) spectra with 300 femtosecond resolution were recorded. As shown in Figure 2, the first 100 ps of spectral evolution are dominated by SE features, with an end-of-pulse spectrum peaking at 512 nm that eventually red-shifts to 545 nm, progressively leading to the onset of GSB features below 480 nm. It is, however, worth noticing that the GSB signal, clearly revealed by the progressive red shift of the SE band, is expected to also be present at early times, although hidden by the overlap with the SE spectral features, and may therefore alter the blue part of the shape of the TA spectra. The 100 ps spectrum then decays, with the formation of a positive signal in the region 600–700 nm, which lasts for longer times and can be attributed to triplet absorption [82].

The last decay can be fitted by a lifetime of ca. 4 ns (Figure S9 in Supplementary Materials), which well matches the measured fluorescence lifetime, supporting the attribution of singlet depopulation with formation of the triplet. On shorter time scales (0–200 ps) a more complex kinetic behavior is observed (Figure S9), with formation and decay of the signal in ca. 1 ps and 25 ps, respectively. This reflects the time evolution of the SE spectrum on short scales, which reveals specific interactions of the excited coumarin dye with the solvent. For comparison with the computational analysis, selected stimulated emission spectra have been extracted from the transient matrix and compared with the simulated profiles, as described in the next sections.

### 2.2. Simulated Spectra

#### Steady-State

**Figure 3:**
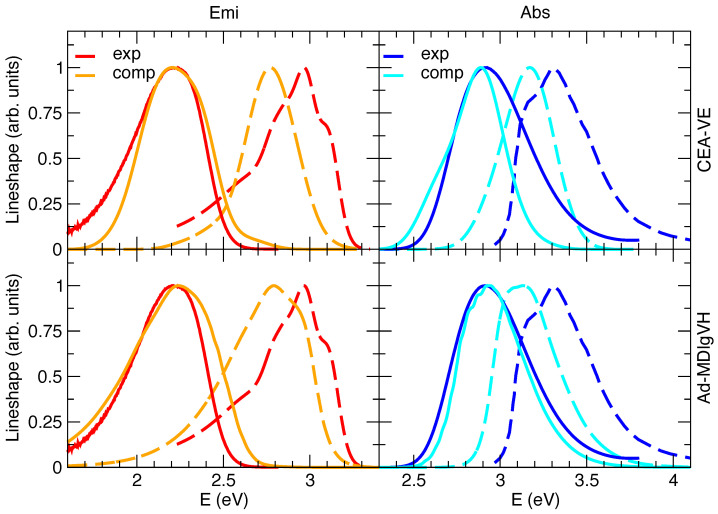
Absorption (abs, right) and emission (emi, left) spectra of C153 in vacuo (dashed lines) or methanol (solid lines) at 300 K, experimentally measured (blue and red lines) or computed (cyan and orange lines) by CEA-VE (top panels) and Ad-MD|gVH (bottom panels). To ease the comparison of the spectral shapes with the experimental signals measured in solution, all computed absorption and emission spectra were shifted by −0.23 eV, accounting for the solvent through model **II**.

The steady-state absorption and emission spectra in methanol were simulated with either the CEA-VE or the Ad-MD|gVH approach, discarding the alternative static FC|VH vibronic calculations. In fact, despite the solvent inhomogeneous broadening can be introduced in FC|VH calculations through Gaussian convolutions (whose width can be computed, for instance, with a continuum model [59]), this strategy actually assumes that solute and solvent motions are completely independent [25], a rather drastic approximation for a polar dye such as C153 surrounded by protic methanol molecules. Moreover, with respect to harmonic approaches such as FC|VH, protocols based on accurate QMD-FFs and MD sampling, such as CEA-VE or the present MQC approach, can also capture the effect on the spectrum of the flexibility of the molecular ring, which is in turn affected by the exchange of energy between solute and solvent. Preliminary simulations in gas phase (see Section E.1 in the Supplementary Materials for details) confirm the good performance of the quantum–classical partition adopted in the Ad-MD|gVH method. Namely, the nine degrees of freedom treated classically correspond to the CF${}_{3}$ torsion and combination of dihedral angles involving N or O${}_{a}$ atoms (see Figure S1 for atomic labels). Moreover, as it appears in Figure 3, the simulated spectra in vacuo already highlight the differences between Ad-MD|gVH and CEA-VE, mainly due to the quantum effects neglected in CEA-VE.

**Figure 4:**
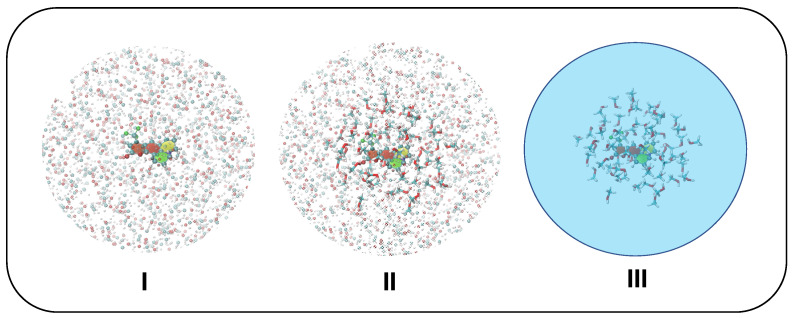
Solvation schemes adopted in CEA-VE and Ad-MD|gVH protocols: model **I**, all solvent atoms within a 18 Å radius from C153 geometrical center are accounted for as point charges (shown with shaded spheres); model **II**, all solvent molecules within a 6 Å radius from C153 geometrical center are accounted for at full QM level (displayed with sticks), while all solvent atoms lying in between 6 and 18Å are still included as point charges; model **III**, all solvent molecules within 6 Å are included at full QM level, whereas the rest of the solvent is accounted for with PCM.

As briefly mentioned in Section 3.1, the solvent molecules surrounding C153 were included in the frames extracted along the MD trajectories according to the different schemes sketched in Figure 4 and outlined in the following:

**I.**  Only the solute is accounted for at QM level, while all methanol molecules within a radius of 18 Å with respect to C153’s geometrical center are treated at MM level through the QMD-FF parameters. Van der Waals terms are only used for water molecules within the first solvation shell while Coulomb terms are considered for all water molecules in the model.
**II.**  The solute and all methanol molecules within a 6 Å radius with respect to C153’s geometrical center are accounted for at QM level, and all methanol molecules found in between 6 Å and 18 Å from C153’s geometrical center are also included in the frame as point charges.
**III.**  The solute and all methanol molecules within a 6 Å radius with respect to C153’s geometrical center are accounted for at QM level, whereas PCM is employed for the rest of the solvent.

It is worthwhile to stress that the effects of the mutual polarization of the solute and the solvent are not included in the MD, since charges are frozen, and a QM/MM dynamics would be required, ideally with a polarizable FF. Notwithstanding this, application of Ad-MD|gVH allows these effects to be introduced on the vibronic Hamiltonians that describe the motion of the stiff degrees of freedom. In fact, according to all the three models listed above, these Hamiltonians are built with QM calculations of energies, forces and Hessians that take into account the solute polarization due to the instantaneous position of the solvent. In addition, when models **II** or **III** are employed, such vibronic Hamiltonians also account for the solute/solvent mutual polarization, either considering only the first solvation sphere (model **II**) or also the average impact of the outer spheres (with PCM, model **III)**. We note that models **II** and **III** can be safely afforded when computing the vertical transition energies and oscillator strengths, as required by both CEA-VE and Ad-MD|gVH, but for the latter approach both solvation schemes become highly demanding, because also the Hessian matrix in the two states is needed for each frame. To limit the computational burden, the calculation of the Hessian matrix was carried out with model **I** only, while transition energies were also evaluated with models **II** and **III**.

In previous sections experimental spectra were reported in the wavelength domain adopted in the measurements. Conversely, since hereafter we focus on the performance of the computed spectra, comparisons will be presented in the energy domain (eV), where it is more straightforward to analyze inaccuracies on vertical transition energies and on the spacing of the vibronic bands. This is done by transforming the quantum distribution of emitted photons Φ(λ) in the frequency domain (Φ(ω)) [83,84]. Moreover both absorption and emission spectra are reported as lineshapes ($L^{abs}\left(\omega \right)=\omega^{-1}\epsilon \left(\omega \right)$ and for spontaneous emission $L^{emi}\left(\omega \right)=\omega^{-3}\Phi \left(\omega \right)$), so that both quantities are directly proportional to the dipole strengths of the underlying vibronic transitions [59,83]. When comparing absorption and emission, the usage of lineshapes is particularly attractive because they are mirror symmetric in the limiting case of simply displaced normal modes [59,85].

Figure 3 reports the computations with model **II**, whereas model **I**’s and **III**’s predictions are given in Figures S7 and S8 in the Supplementary Materials. By comparing computed and experimental spectral shapes, the results confirm the trends found in the gas phase and displayed in the Supplementary Materials in Figure S6: notwithstanding that Ad-MD|gVH does not adopt any phenomenological broadening, it predict shapes in much better agreement with experiment than CEA-VE, which instead uses a phenomenological Gaussian with HWHM = 0.1 eV. Moving to the position of the spectra, one can notice that, in order to overlap the computed and experimental spectra in methanol, all the computed spectra in Figure 3 are displaced by −0.23 eV. On the one hand, considering that the position of the computed spectra in the gas phase is rather accurate (the error is ∼0.07 eV), we can estimate that the computed error on the solvent shift from gas phase to methanol is ∼0.15 eV. This inaccuracy is emphasized visually in Figure 3, where, after applying the shift necessary to overlap the spectra in solution, the computed spectra in the gas phase appear substantially red-shifted with respect to the experiment. On the other hand, our approach reproduces quite well the Stokes shift, as indicated by the very good overlap between computed and experimental spectra obtained by shifting both absorption and emission of the same quantity. Interestingly, in the Supplementary Materials we show that application of model **I** leads to a blue-shift of both absorption and emission spectra (but by different extents), worsening their absolute position and also the prediction of the Stokes shift. This finding confirms the importance of a proper consideration of the mutual solute/solvent polarization [34,69,86]. On the contrary, model **III** leads to a very good reproduction of the solvent shift from gas to methanol in absorption, whereas it introduces a ∼0.10–0.15 eV error on the Stokes shift, probably reminiscent of some asymmetry in the description of absorption and emission with PCM and TD-DFT, as discussed by some of us in Ref. [59]. Therein, it was indeed shown that a good reproduction of the spectral shapes of C153 in aprotic polar solvents could also be obtained adopting PCM and computing the solvent inhomogeneous broadening according to Marcus’ theory, from the solvent reorganization energy computed with state-specific PCM implementation. The Stokes shift, however, showed a remarkable inaccuracy of ∼0.4 eV (results in acetonitrile). Beside these differences, PCM calculations, in the implementations available in distributed packages, cannot describe the dynamics of solvent rearrangement after excitation and therefore such an implicit model is not suited to compute TR spectra, which instead can be tackled by our Ad-MD|gVH approach, as shown in the next section.

### 2.3. Simulated Time-Resolved Emission Spectra

The analysis of the experimental data indicates that in the first 100 ps the TA spectra are dominated by SE, apart from a small GSB on the very high-energy wing of the band, and that on this time scale the emitting state does not decay either to the triplet or to the ground state. It is therefore possible to compare the lineshapes of the experimental TA spectra (turned to positive) and of the computed TR SE spectra. Furthermore, in this case, the comparison is made between their lineshapes, obtained by dividing the spectra by ω.

**Figure 5:**
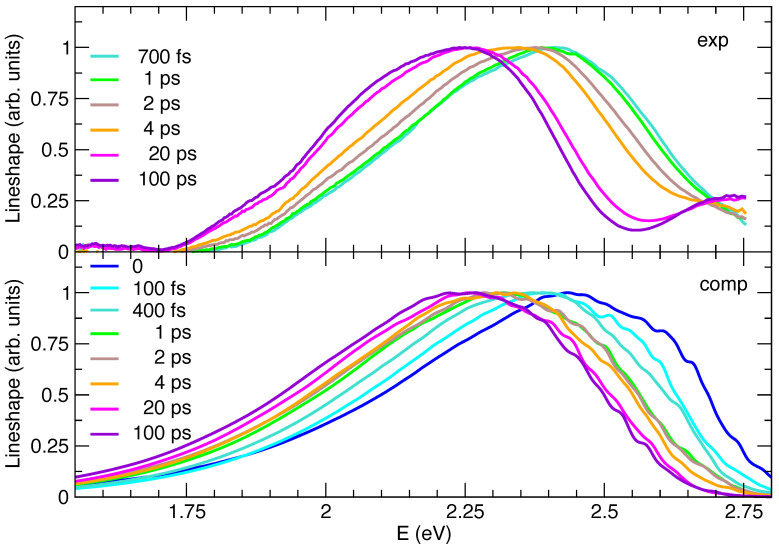
Comparison between lineshapes of the experimental transient absorption spectra (exp, top panel) and the computed time-resolved emission spectra (comp, bottom panel). All signals refer to the C153 dye at 1 atm and 300 K, in methanol solution, which in the computed spectra is accounted for through model **II**. Note that, to ease the comparison, all experimental signals were turned to positive and, in line with steady-state emission, all computed spectra were shifted by −0.23 eV.

Figure 5 shows that, as far as the spectral shapes are concerned, simulations generally agree with experiments in capturing the asymmetry of the spectra, with a longer red wing. It can be noted that, in TA spectra, SE features are superimposed with GSB and excited state absorption. The latter contribution can explain the reduced intensity of the SE red wing in the experimental spectra with respect to the simulated SE ones in Figure 5. A more detailed analysis reveals that computations also reproduce well the slight differences between spectral signals at early and long times. Nonetheless, some minor discrepancies, observed for steady-state emission (see Figure 3), also affect transient spectra, being in some cases accentuated. At all times, the computed spectra are slightly too broad and the predicted left skewed asymmetry is too large with respect to experiments. In this respect, it is interesting to notice that the computed TR emission at 100 ps is superimposed to the steady-state one, whereas, on the contrary, the red-wing of the experimental TR emission at 100 ps is less pronounced than in the steady-state emission (Figure S10 in the Supplementary Materials). It can be noted that, in TA spectra, SE features are superimposed with GSB and excited state absorption.

In order to ease the comparison between the predicted and measured dynamics of the spectral peak positions, in Figure 5 all the Ad-MD|gVH spectra are displaced by −0.23 eV, i.e., the same amount necessary to match the computed and experimental steady-state emission. The experimental general trend is nicely reproduced, yet, for a more detailed analysis, it should be noticed that at early times the comparison is made more difficult by the fact that the experimental signal is measured with a finite time resolution (∼300 fs), whereas computed ones are obtained assuming an excitation that is a δ in time. For a more proper comparison, to simulate the finite-resolution of experiments, it would be therefore necessary to convolute the computed spectra with a Gaussian function of time. This procedure would, however, be too computationally demanding, as it would be required to first compute the spectra on a dense time grid. For these reasons we start from an analysis at long times and then we move to early times, where the comparison is more elusive. The maxima of the TR experimental and computed spectra are given in Table 2, where it appears that their position monotonically shifts in time toward the red, with a single exception in the spectra computed at 4 ps, where the maximum is moving in the opposite direction.

This is simply due to the noisy and very flat shapes of the spectra in the maximum region, which makes its precise determination not very meaningful, as confirmed by the fact that the center of gravity of the spectra at 2 and 4 ps is practically indistinguishable. The comparison between simulated and experimental spectra is very good going back from 100 ps until 4 ps and correctly reproduces the fact that the solvent reorganization is concluded after 20 ps. On the contrary, before 4 ps some discrepancies emerge: between 2 and 4 ps the shift of the experimental spectrum is significant, whereas it is negligible according to simulations. This finding suggests that on this timescale the solvent dynamics are predicted to be too fast in the simulations. This conclusion is confirmed by the analysis at 1 ps: in Figure 5 the simulated spectrum almost perfectly overlaps with the one at 2 ps and is similar to the one computed at 4 ps, whereas the experimental signal shows non-negligible differences with respect to the one registered at 2 or 4 ps. It should be recalled here that, as mentioned in the previous sections, the measured spectra include to a certain extent a GSB contribution, which is not accounted for in simulations. It is in fact also possible that at early times the high-energy wing of the SE component of the experimental TA spectra is damped by GSB bleaching.

In the sub-ps timescale the uncertainty due to the experimental time-resolution becomes even more significant, making a comparison less robust.

However, quite interestingly, computations also give us access to an ideal t=0 spectrum, after an instantaneous pump, information that is not accessible in the experiments due to the finite time resolution. Comparison between simulated spectra at *t* = 0 to 100 fs suggest that, even in this very short time, the solvent dynamical response gives rise to a significant red-shift ∼0.06, which is unobserved in the experiments (solvent dynamics is already activated during the excitation process). It is also noteworthy that, at these early times, the shape of the computed spectra is also moderately different from at later times, with a more intense shoulder at high energies, which is not observed in the experiments. Since, in our computed protocol, the molecular stiff vibrations are considered to relax instantaneously to a Boltzmann distribution (an approximation discussed below), we can conclude that such a high-energy shoulder can be traced back to a solvent effect. Moreover, we can speculate that this is likely connected to a fraction of solvent configurations which are particularly unfavorable to the excited state, which, for this very same reason, are quickly (already in ∼400 fs) abandoned during the dynamics following the excitation. Coming to the approximation that the solute degrees of freedom, treated at quantum (vibronic) level, are always thermally equilibrated (which is intrinsic in the adoption of analytical thermal correlation functions in TD vibronic computations [87]), we notice that such stiff modes are usually characterized by high frequencies and therefore thermal excitation is in any case very moderate. Indeed, Figure S11 in the Supplementary Materials confirms that Ad-MD|gVH spectra obtained assuming a temperature of 0 K for the vibronic computations are rather similar to the one presented here. Although thermal excitation is not an issue, it is on the contrary possible that, at very early times, our approach misses some out-of-equilibrium effect due to the excess of energy deposited by the photoexcitation on some high-frequency mode. Such an effect would be expected to produce spectra that are even broader than the thermally excited one [88]. For the specific case of C153, such a broadening is not observed even in the experiment (it should arise in the comparison of the spectrum in the long-time limit, with those recorded at very early times). However, in such a fast time regime (<1 ps), neglecting the aforementioned contribution of the GSB in the interpretation of the TA as a pure SE might lead to an underestimation of the spectral width and an artificial red-shift of its maximum. It is therefore confirmed that the comparison of theory and experiments at such times can be only qualitative.

### 2.4. Solvation Structure during Time-Resolved Emission

**Figure 6:**
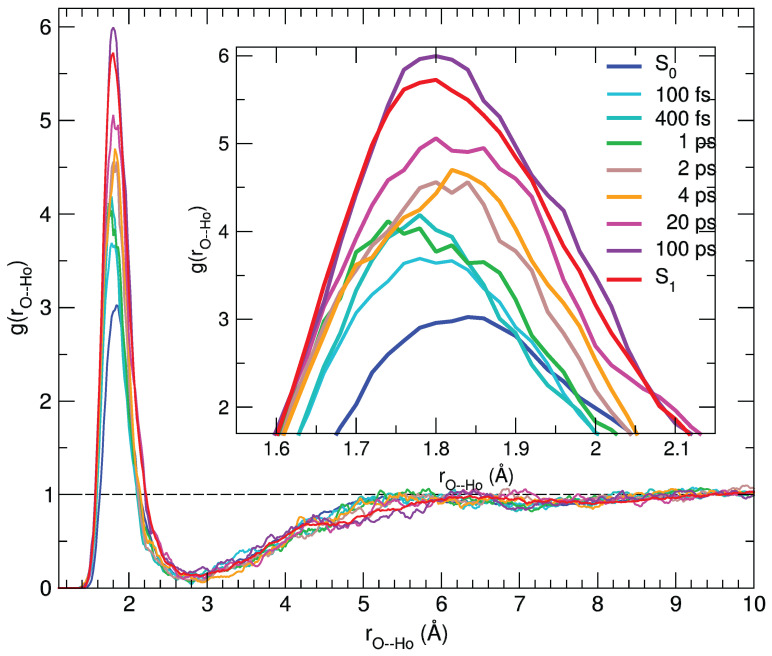
Pair correlation functions between the (C=)O atom and the hydroxyl proton (Ho) of the solvent, computed for C153 at different time intervals and averaged over 100 snapshots.

The agreement between the calculated and experimental transient spectra confirms the reliability of the MD sampling, which can therefore be confidently exploited to gain a deeper insight in the microscopic origin of the observed spectral features. Indeed, the dynamical response of the solvation structure to the electronic excitation of the C153 dye can be first analyzed in terms of radial distribution functions g(r${}_{\alpha \beta }$) between specific coumarin atoms (α) and the hydroxyl proton of the methanol solvent (β), computed by averaging along the 500 nonequilibrium MD runs at the time intervals selected for the transient spectra simulation. By looking at Figure S9 in the Supplementary Materials, it is evident that the most significant changes take place in the vicinity of the carbonyl group. Figure 6 shows the g(r${}_{O-H_{O}}$) radial distribution function of the hydroxyl proton (H${}_{O}$) around the (C=)O atom of the dye, either computed along the nonequilibrium runs or obtained by averaging the equilibrium S${}_{0}$ and S${}_{1}$ trajectories employed for the steady-state absorption and emission spectra, respectively.

As evidenced in the inner panel, the remarkable response upon excitation is clearly connected to the charge flux toward the O atom, which in turn strengthens its interaction with the closest methanol protons, resulting in a g(r${}_{O-H_{O}}$) first peak of increasing intensity and shifting to smaller distances. Interestingly, such a process covers the whole 100 ps range, where the average methanol-C153 hydrogen bond is first shifted to shorter distances in the sub-picosecond regime and thereafter (2–100 ps) slightly relaxed to let more solvent molecules enter the first solvation shell, as indicated by the peak growing intensity. A further analysis of the reorganization of the solvent surrounding the excited solute in the first solvation shell can be carried out resorting to orientational correlations functions, again exploiting the statistical ensembles created at different time intervals across the nonequilibrium MD runs. By inspecting the variation of the atomic charges on the C153 sites upon excitation (see also Table S2 in the Supplementary Materials), it appears that the atoms experiencing the largest change are N (Δ q = + 0.09 e) and the carbon atom of the carboxyl group, Co (Δ q = −0.10 e, see Figure S1 for the atom labels definition). As already noted, this charge flux is consistent with the direction of the charge transfer induced by the electronic transition [59,72,74]. We defined one unit vector for C153, oriented from the Co atom to the N atom (${\hat{u}}_{1}$), and another for the solvent, from the O atom to the Ho proton (${\hat{u}}_{2}$), eventually computing the function $h^{110}\left(r\right)$, provided by the rotational invariant expansion [89]:

(1) $$h^{110}\left(r\right)=\frac{\int h\left(12\right)\Phi^{110}\left(12\right)d\Omega_{1}\Omega_{2}}{\int {\left[\Phi^{110}\left(12\right)\right]}^{2}d\Omega_{1}\Omega_{2}},$$

**Figure 7:**
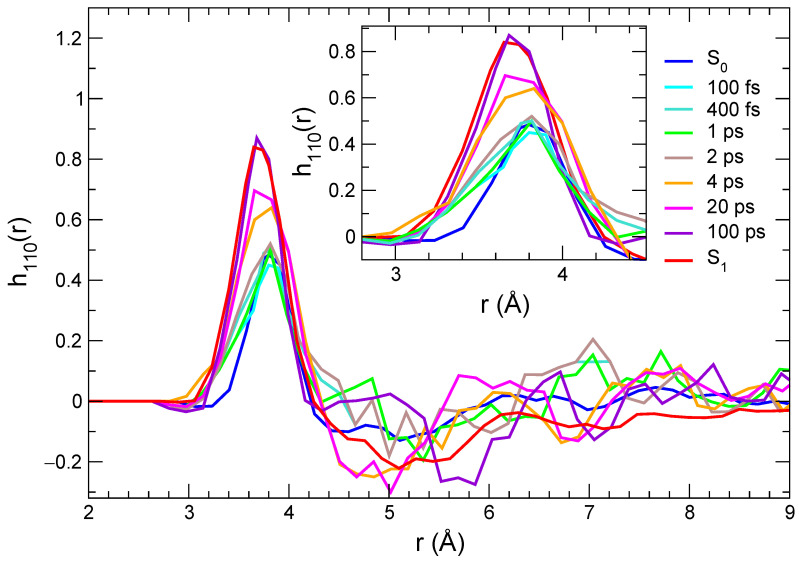
Orientational correlations describing dipolar solute–solvent interactions computed at different times, and averaged over the nonequilibrium trajectories and for the two equilibrium trajectories (solute in its ground and in its excited state).

Here

(2) $$\Phi^{110}\left(12\right)={\hat{u}}_{1}\cdot {\hat{u}}_{2},$$

where $\Omega_{i}$ are the angular variables and $\Phi^{110}$ is the rotational invariant function depending on dipolar correlations between molecules 1 (C153) and 2 (methanol). By averaging over the 500 nonequilibrium trajectories, we obtained, for each time interval considered for the transient spectra, the curves displayed in Figure 7. In keeping with the radial distribution function analysis, the results achieved by averaging along the S${}_{0}$ and S${}_{1}$ equilibrium trajectories are also reported in the same figure.

Although the $h^{110}\left(r\right)$ functions need more statistics than the radial distributions, thus leading to noisier results at distances for which smaller densities are observed, the behavior in the first solvation shell, corresponding to the first peak, can be qualitatively discussed. As a general remark, the first peak is positive, which is consistent with a parallel orientation of the ${\hat{u}}_{1}$ and ${\hat{u}}_{2}$ vectors, and correlations increase strongly with time. An additional effect that can be observed is that such peaks move to slightly shorter distances and become broader, which is consistent with a more populated solvation shell in the excited state compared with the ground state and with stronger local solute–solvent interactions. One interesting difference is observed here compared with the time evolution of the radial distribution functions. As a matter of fact, orientational correlations do not increase strongly before 4 ps; then they evolve rapidly, until a distribution that is very close to that computed in the equilibrium S${}_{1}$ state is observed (100 ps). These results point to a response mechanism in which the solvent reacts to the local perturbation due to the solute excitation by quickly (up to 2 ps) getting closer to those sites that are sensitive to specific interactions (e.g., along the hydrogen bond) and then by reorienting (on a > 2 ps time scale) in order to maximize local dipolar interactions. This is consistent with the interpretation of the solvation response in methanol based on MD simulations, according to which an important channel for the solvent relaxation is provided by the reorientation of the O-H axis [90,91].

## 3. Materials and Methods

### 3.1. Computational Protocols

#### 3.1.1. Steady-State Absorption and Emission Spectra

**Figure 8:**
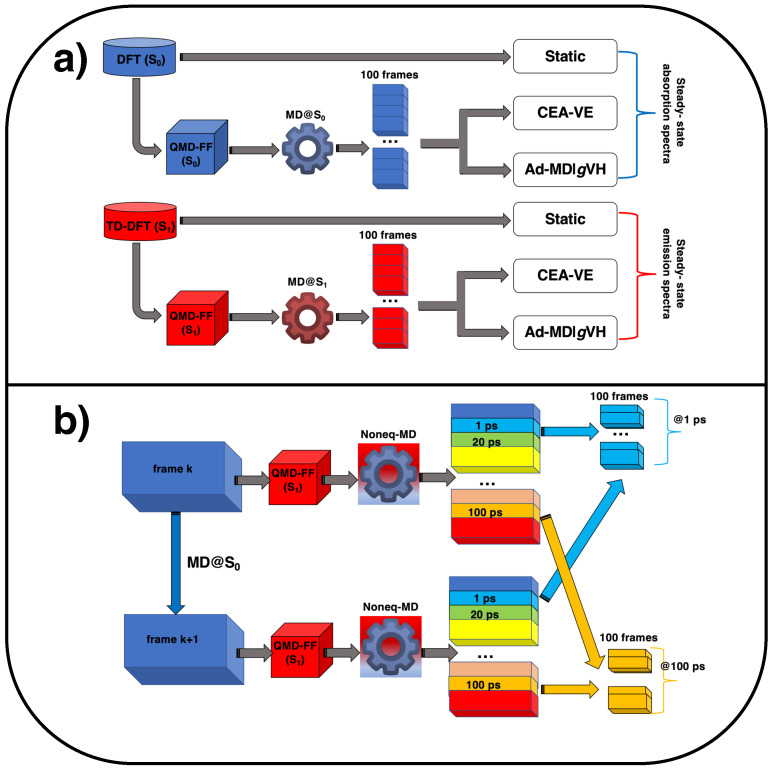
Pictorial sketch of the computational protocols applied in this work to simulate (**a**) steady-state (blue, top) and emission spectra (red, bottom) and (**b**) time-resolved emission spectra, exploiting nonequilibrium MD.

As displayed in Figure 8a, the steady-state absorption and emission spectra of the coumarin C153 dye in methanol were computed in this work according to three different strategies, whose details are briefly outlined in the following.

(i) Static vibronic approach
  This approach accounts for the effect of intra-molecular vibrations at quantum level and it is based on a single molecular structure, namely the optimized geometry, which is separately considered in its ground or first electronic excited state, for the simulation of absorption or emission spectra, respectively. Since it does not require MD to explicitly simulate the system dynamics, it will be hereafter referred to as “static”. The optimized, state-specific geometries were employed together with their corresponding Hessian matrices to build up harmonic model PESs and compute the vibronically resolved static spectra with the vertical Hessian (VH) model, which accounts for the effects of normal mode mixings (Duschinsky rotation) [92] on the spectra and it is based on a Taylor expansion of both the initial- and final-state PESs at the initial-state geometry up to the quadratic terms. Moreover, the Franck–Condon (FC) approach was also applied, an approximation fully adequate due to the brightness of the electronic transition.
(ii) Classical ensemble average of vertical energies (CEA-VE) approach
  This approach is based on the FC classical principle and exploits classical MD to sample the configurational space in the initial state. MD trajectories were first carried out with QMD-FFs, specifically tailored for the target molecule in S${}_{0}$ and S${}_{1}$. Absorption and emission spectra in gas phase and in methanol were thereafter computed over 100 snapshots sampled along equilibrated MD trajectories, obtained in vacuo and on the solvated C153 dye, with either the S${}_{0}$ or S${}_{1}$ QMD-FFs. The final spectral shape is retrieved by a classical ensemble average (CEA) of the vertical excitations (VE) computed at each extracted snapshot.
(iii) Ad-MD|gVH approach
  This is a MQC method designed to re-introduce the quantum effect of the relevant molecular vibrations on top of a classical MD sampling of the remaining nuclear DoFs. In practice, for each of the $N_{conf}$ snapshots extracted from either the S${}_{0}$ or S${}_{1}$ MD runs, a frame-specific vibronic spectrum is also obtained according to the TD formulation, based on analytical correlation functions [59,87,93,94,95,96,97], implemented in the FCclasses3.0 software [98,99]. The latter code exploits QM energies, gradients and Hessians, for the initial and final electronic states, and a partition of the nuclear DoFs into soft and stiff modes [31,99]. At each snapshot α, vibronic spectra are computed with the so-called generalized VH (*g*VH) model, building up reduced-dimensionality harmonic potential energy surface (PES) for the stiff modes (*r*), obtained by projecting out the soft modes from gradients and Hessians. The vibronic signals specific to each different snapshot are eventually averaged to obtain the final Ad-MD|gVH spectrum, $L^{MQC}(\omega$), which reads
  (3) $$\begin{array}{c} L^{MQC}\left(\omega \right)=\frac{1}{N_{conf}}\sum_{\alpha =1,N_{conf}}L_{r}^{\alpha ,Q}\left(\omega \right) \end{array}$$
  where in $L_{r}^{\alpha ,Q}\left(\omega \right)$ the subscript *r* indicates that the spectrum is computed including the contributions of the fast stiff coordinates only, and the superscripts *Q* and α that it is computed at quantum vibronic level (*Q*) specifically for the α configuration of the soft modes.

#### 3.1.2. Time-Resolved Emission Spectra

As shown in Figure 8b, the TR emission spectra have been computed, within both the CEA-VE and Ad-MD|gVH frameworks, along the snapshots extracted from nonequilibrium MD runs. Concretely, the $N_{conf}$ uncorrelated configurations, sampled from the equilibrium trajectory obtained for C153 in methanol with the ground-state QMD-FF and employed in the absorption steady-state spectrum, were now used as starting points for 500 separate nonequilibrium runs, where the sudden electronic excitation is mimicked by the change of FF, as sketched in Figure 8b. Snapshots $\beta_{t}$ are thereafter extracted from each of the nonequilibrium trajectories at selected time intervals (*t*), namely 100 fs, 400 fs, 1 ps, 2 ps, 4 ps, 20 ps and 100 ps, and successively grouped together to build statistically meaningful ensembles for each considered *t*. The computed transient spectra are eventually reported as “stimulated emission”. Focusing on the Ad-MD|gVH spectrum, its mathematical expression is analogous to the one for the steady-state case (see Equation (3), where α = $\beta_{t=0}$):

(4) $$\begin{array}{c} L_{t}^{MQC}\left(\omega \right)=\frac{1}{N_{conf}}\sum_{\beta_{t}=1,N_{conf}}L_{r}^{\beta_{t},Q}\left(\omega \right) \end{array}$$

where, however, the index *t* indicates that the spectra are computed generating a different *g*VH model at each time interval *t* for each of the nonequilibrium snapshots $\beta_{t}$.

### 3.2. Computational Details

All QM calculations were carried out in this work with the Gaussian16 package [100], resorting to density functional theory (DFT) or its time-dependent implementation (TD-DFT), making use of the PBE0 functional with the 6-31G* basis set; this combination was already tested in the past for C153 [59,74]. The static spectra were all obtained, either at FC|VH or FC|AH (adiabatic Hessian) [101], with the FCclasses3.0 code [99,102].

**Figure 9:**
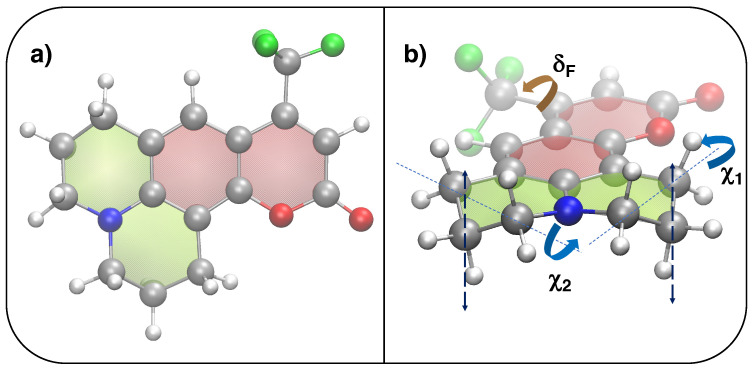
Top and side views of the chemical structure of coumarin C153. (**a**) Optimized QM structure (S${}_{0}$, PBE0/6-31G*): silver, red, blue, white and green spheres are employed for C, O, N, H and F atoms, respectively, whereas red and green shaded surfaces indicate aromatic or aliphatic rings. (**b**) Definition of flexible coordinates: $\delta_{F}$ (brown) governing the CH${}_{3}$ rotation and $\chi_{1(2)}$ (blue), ruling the CH${}_{2}$ out-of-plane motion highlighted with the dashed lines.

The QMD-FFs employed in this work are parameterized based on a set of QM calculations purposely carried out on the C153 coumarin (see Figure 9), either in the S${}_{0}$ or $S_{1}$ electronic state.

Although all details of the parameterization procedure are reported in the Supplementary Materials or in the original papers [67,68], a brief outline of the adopted strategy is given in the following. The two for S${}_{0}$ and S${}_{1}$ intramolecular QMD-FFs were separately parameterized, with the Joyce package [103], using, respectively, the DFT and TD-DFT training database described in the Supplementary Materials. Concretely, all C153 intramolecular parameters appearing in Equations (S5)–(S8) were obtained through a least square minimization of the standard Joyce objective function [67,68]:

(5) $$\begin{array}{c} I^{intra}=\sum_{g=0}^{N_{geom}}W_{g}{\left[U-E_{intra}^{QMD-FF}\right]}_{g}^{2}+\sum_{K\leq L}^{3N-6}\frac{2W_{KL}^{\prime \prime }}{\left(3N-6)(3N-5\right)}{\left[H_{KL}-\left(\frac{\partial^{2}E_{intra}^{QMD-FF}}{\partial Q_{K}\partial Q_{L}}\right)\right]}_{g=0}^{2} \end{array}$$

where $N_{geom}$ is the number of the conformations *g* considered in the QM training database (see Supplementary Materials), $Q_{K}$ is the Kth normal coordinate, $U_{g}$ is the QM energy computed in the gth geometry and *H* the Hessian matrix at the absolute minimum (g=0). $W_{g}$ and $W_{KL}^{\prime \prime }$ are the standard [67,104] weighting factors for energies and Hessian, respectively. Finally, the use of classical MD methodologies with the ground/excited states electrostatic potential partial charges for equilibrium/nonequilibrium trajectories, respectively, is the standard approach for this kind of simulation, when rather long timescales need to be achieved [65,66]. Following this approach, to refine C153–methanol interactions, the point charges that contribute to the inter-molecular QMD-FF energy term (see Equations (S3) and (S4) in the Supplementary Materials) were derived, for both S${}_{0}$ and S${}_{1}$ FFs, through the RESP procedure from the DFT or TD-DFT electronic density, consistently computed at PBE0/6-31G* level, accounting for the methanol solvent at PCM level (for S1 with the linear response implementation in the equilibrium regime) [105].

All classical MD simulations were carried out with the gromacs code [106], in the NVT and NPT ensembles, respectively, for the isolated C153 dye (gas phase) and a solvated system, consisting of one coumarin embedded in ∼1000 methanol molecules. For the isolated molecule, two 10 ns trajectories at 383 K were produced using either the S${}_{0}$ or the S${}_{1}$ QMD-FF, storing dye’s conformations every 100 ps. As far as the solvated system is concerned, both S${}_{0}$ and S${}_{1}$ equilibrium runs were simulated for 10 ns, extracting frames every 20 ps. For S${}_{0}$, the extracted frames were thereafter used to run 500 separate nonequilibrium MD runs for 100 ps each, storing the system coordinates at selected time intervals *t* from every resulting trajectory, hence building a reliable configurational ensemble to simulate transient spectra. Further details on MD runs can be found in Section D of the Supplementary Materials. Steady-state and time-resolved spectra were computed averaging over 100 configurations, while all 500 nonequilibrium runs were used to evaluate structural properties over the nonequilibrium dynamics. From the snapshots used to compute the spectra, solute and solvent were described at QM/MM level, according to different solvation models discussed in detail in the next sections.

For each frame collection, i.e., those extracted from the ground and excited state equilibrium trajectories and those sampled along every nonequilibrium run, the spectra were computed according to both CEA-VE and Ad-MD|gVH approaches. In the former case, the vertical transition energies and oscillator strength computed for each frame were first convoluted with a Gaussian function with HWHM = 0.1 eV. When adopting the Ad-MD|gVH protocol, the vibronic spectra computed along the extracted frames (in gas phase or methanol) have been obtained treating nine degrees of freedom that correspond to combinations of several dihedrals as classical degrees of freedom (DoF) and all other internal DoF as quantum coordinates. The classical modes describe the rotation of the CF${}_{3}$ group, some flexibility involving the carbon adjacent to the oxygen in the ring and the pyramidalization of the nitrogen. With this choice, the reduced-dimensionality Hessians used to build up vibronic Hamiltonians exhibit all real frequencies, for almost all snapshots. Nevertheless, the Ad-MD|gVH approach accounts explicitly for all possible broadening mechanisms and, in principle, the use of a phenomenological Gaussian is not required, yet the finite ensemble average may still display some noise. According to what is proposed in ref. [31], such noise was significantly reduced by convolution with a narrow Gaussian (HWHM = 0.01 eV) without affecting the general shape and width of the spectra.

### 3.3. Experimental Set Up

The spectroscopic investigations for C153 were carried out in spectrofluorimetric grade methanol (MeOH) (Merck Uvasol^®^) and iodoethane (C${}_{2}$H${}_{5}$I) (Alfa Aesar, Haverhill, MA, USA). Perkin-Elmer Lambda 950 spectrophotometer was used to record the absorption spectra. Photoluminescence experiments were carried out by putting the C153 solutions into 1 cm fluorimetric Suprasil quartz gas-tight cuvettes. Uncorrected emission spectra were collected by an Edinburgh Instruments FLS920 spectrometer equipped with a Peltier-cooled Hamamatsu R928P photomultiplier tube (185–900 nm) and a 450 W xenon arc lamp as the excitation light source. All emission spectra were corrected via a calibration curve supplied with the instrument. The photoluminescence quantum yield ($\phi_{em}$) of C153 in methanol was determined from the corrected emission spectra by using coumarin 153 ($\phi_{em}=0.53$) in ethanol (Merck Uvasol^®^) solution as reference [107]. The nanosecond (ns) excited state lifetimes (τ) were measured by the time-correlated single photon counting (TCSPC) technique with a HORIBA Jobin Yvon IBH FluoroHub equipped with a pulsed NanoLED ($\lambda_{exc}=373$ nm) excitation source and a TBX-05C Picosecond Photon Detection Module (300–850 nm) as the detector. The nanosecond luminescence decay profiles were analyzed with the DAS6 Decay Analysis Software, and the quality of the fit was assessed with the $\chi^{2}$ value (close to unity) and with the residuals randomly distributed along the time axis. Microsecond (μs) lifetimes were collected through the Edinburgh Instruments FLS920 spectrometer equipped with an Edinburgh μF 920H pulse excitation light source at 373 nm. Low temperature luminescence spectra (77 K) were recorded by 2 mm inner diameter quartz tubes (sample inside) fitted into a special quartz cold finger Dewar filled with liquid nitrogen. Experimental uncertainties are estimated to be ±8% for τ determinations, ±20% for $\phi_{em}$, and ±2 nm and ±5 nm for absorption and emission peaks, respectively. Pump–probe transient absorption measurements were performed with an Ultrafast Systems HELIOS (HE-VIS-NIR) femtosecond transient absorption spectrometer by using a fiber optics coupled multichannel VIS spectrometer with CMOS sensor with 1.5 nm intrinsic resolution. As excitation source, a Newport Spectra Physics Solstice-F-1K-230 V laser system, combined with a TOPAS Prime (TPR-TOPAS-F) optical parametric amplifier (pulse width: 100 fs, 1 kHz repetition rate) tuned at 350 nm was used. A sapphire crystal for continuum generation in the visible range (450–800 nm) was employed. The overall time resolution of the system is 300 fs. Air-equilibrated solutions in 0.2 cm optical path cells were analyzed under continuous stirring. The pump energy on the sample was 6 μJ/pulse. Surface Xplorer V4 software from Ultrafast Systems was used for data acquisition and analysis. The 3D data surfaces were corrected for the chirp of the probe pulse prior to analysis.

## 4. Conclusions

In this contribution we have reported a combined experimental and theoretical investigation into the steady-state and transient spectroscopy of the coumarin C153 in methanol, with the aim of applying for the first time a recently developed mixed quantum–classical methodology, the Ad-MD|gVH approach, to the simulation of time-resolved emission spectra. This approach relies on the separation of the nuclear DoFs of the solute+solvent ensemble in soft modes (treated through classical MD based on accurate QMD-FFs) and stiff modes (treated quantum mechanically). The soft and stiff modes are coupled through an adiabatic approximation which considers quantum modes fast enough to rearrange instantaneously to any fluctuation of the soft modes. By combining this methodology with nonequilibrium MD simulations, mimicking the solute–solvent relaxation dynamics after the pump pulse, we have shown it to be capable of reliably predicting the shape and temporal evolution of the transient stimulated emission signal. Moreover, the analysis of the nonequilibrium MD trajectories provided us with atomistic details on the solvent reorganization following the photo-induced intramolecular charge transfer, that we have discussed in terms of pair correlation functions between the carbonyl oxygen atom and the hydroxyl proton of the solvent and orientational correlation functions describing dipolar solute–solvent interactions.

As far as the steady-state spectra are concerned, our results show that, although Ad-MD|gVH does not make use of any phenomenological broadening, it delivered shapes in much better agreement with experiments than those obtained by Gaussian convolution of the CEA-VE transitions computed at extracted configurations along the equilibrium MD runs. Moreover, our approach reproduced quite well the Stokes shift, notwithstanding a small error (∼0.15 eV) on the predicted solvent shift. Moving to the pump–probe spectroscopy, transient absorption spectra with 300 femtosecond resolution were recorded, showing that the first 100 ps of spectral evolution are dominated by stimulated emission signals, with an end-of-pulse spectrum peaking at 512 nm that eventually red-shifts to 545 nm, progressively leading to the onset of ground state bleaching features below 480 nm. The shape of the time-resolved stimulated emission signals was, overall, nicely reproduced by the Ad-MD|gVH method, even if the calculated spectra were slightly too broad and showed a more pronounced asymmetry at low energies when compared to the experimental ones. As discussed, this discrepancy is ascribable to the fact that, in TA spectra, SE features are superimposed with GSB and ESA.

Focusing on the spectral shift induced by the methanol reorganization dynamics, from 100 ps to 4 ps, the simulated spectra were in excellent agreement with experiments and they correctly indicated the end of the solvent response within about 20 ps. At a shorter time-scale, that is before 4 ps, faster solvent dynamics were conversely predicted by simulations, although in the sub-ps timescale the experimental uncertainty due to the time resolution and the contribution of the GSB, and the approximation of an instantaneous thermalization of stiff-modes in the vibronic computations, make the comparison less straightforward. By inspection of the solvent reorganization across the nonequilibrium MD trajectory, one can see that the main solvent response upon excitation is the strengthening of the interaction between the carbonyl oxygen and the closest methanol protons. In more detail, the average hydrogen bond distance is shortened in the first ps, whereas it relaxes back to the original length after 2 ps, yet progressively increasing the population of the first solvation shell. Further mechanistic insights came from the analysis of the orientational correlation functions, which indicate that, after the strengthening/relaxation process of the hydrogen bonds around those sites that are sensitive to specific interactions highlighted by the radial distribution functions, the solvent reacts by reorienting (on a ≈ps timescale) in order to maximize local dipolar interactions. Interestingly, the time range at which the re-orientational process takes place (4–100 ps) coincides with the one where the simulated spectra better agree with the experimental signals, whereas the fast (100 fs–2 ps) local perturbations due to the strengthening of the methanol-C153 hydrogen bonds seem to induce spectral features in the simulations that are either not registered by the experiment or too fast. This last observation calls for future further investigations, to ascertain whether the simulated features are realistic, yet hidden in the experiment or if, instead, a further refinement of the QMD-FF is still needed, in particular in the intermolecular term which governs the solute/solvent interactions.

## Figures and Tables

**Table 1:** Spectral parameters of C153: $\lambda_{max}$ and ϵ for absorption, and $\lambda_{max}$, lifetime τ and yield ϕ for fluorescence and phosphorescence emission. ^a^ In methanol. ^b^ In methanol/ethyliodide (1:1). ^c^ From corrected emission spectra. ^d^ Fluorescence quantum yield, measured with reference to C153 in ethanol as a standard. ^e^ Fluorescence and phosphorescence lifetimes were measured by exciting at 373 nm.

**Absorption** ^a^ (298 K)			
$\lambda_{max}$ (nm)		ϵ (10${}^{4}$ M${}^{-1}$ cm${}^{-1}$)	
221, 266, 424		2.14, 0.77, 1.76	
**Emission** (298 K)			
$\lambda_{max}$ ^c^ (nm)		$\tau_{flu}$ ^d^ (ns)	$\phi_{em}$ ^e^ (%)
537		4.0	42.2
**Emission** (77 K)			
$\lambda_{maxflu}$ ^a,c^ (nm)	$\lambda_{maxphos}$ ^b,c^ (nm)	$\tau_{flu}$ ^a,d^ (ns)	$\tau_{phos}$ ^b,d^ (μs)
497	603, 633	5.8	0.9 (52%), 7.4 (48%)

**Table 2:** Computed (Ad-MD|gVH, left) and measured (right columns) peak maxima (E${}_{max}$, eV) and their time shifts (ΔE${}_{max}$, eV) in time-resolved emission spectra. All data refer to Figure 5, where computed maxima are shifted by −0.23 eV.

Ad-MD|gVH			Experiments		
**Time (ps)**	**E${}_{\max}$ (eV)**	**ΔE${}_{\max}$ (eV)**	**Time (ps)**	**E${}_{\max}$ (eV)**	**ΔE${}_{\max}$ (eV)**
0	2.43	0.00			
0.1	2.39	−0.04			
0.4	2.37	−0.06	0.7	2.41	0.00
1	2.32	−0.11	1	2.39	−0.02
2	2.28	−0.15	2	2.38	−0.03
4	2.31	−0.12	4	2.34	−0.07
20	2.26	−0.17	20	2.26	−0.15
100	2.22	−0.21	100	2.24	−0.17

## Data Availability

The data presented in this study are available on request from the corresponding authors.

## Supplementary Materials

The following supporting information can be downloaded at: https://www.mdpi.com/article/10.3390/molecules28093910/s1, Further details on QMD-FF parameterization and MD simulations, steady-state and transient spectra and supplementary Figures S1–S12 [31,59,67,68,103,104,105,108,109,110,111,112,113,114].

## Abbreviations

The following abbreviations are used in this manuscript:

MQC	Mixed quantum–classical
MD	Molecular dynamics
TR	Time-resolved
GSB	Ground-state bleaching
SE	Stimulated emission
ESA	Excited-state absorption
UV-Vis	Ultraviolet–visible
TD-DFT	Time-dependent density functional theory
QMD-FF	Quantum mechanical derived force field
Ad-MD|gVH	Adiabatic molecular dynamics generalized vertical Hessian
DOF	Degree of freedom
C153	Coumarin 153
S${}_{0}$	Ground electronic state
S${}_{1}$	First excited state
VH	Vertical Hessian
PES	Potential energy surface
FC	Franck–Condon
CEA-VE	Classical ensemble average of vertical energies
AH	Adiabatic Hessian
QM	Quantum mechanics
MM	Molecular mechanics
HWHM	Half width at half maximum
PCM	Polarizable continuum model
