# Supplementary material for: Non-Phenomenological Description of the Time-Resolved Emission in Solution with Quantum–Classical Vibronic Approaches—Application to Coumarin C153 in Methanol
Source: Molecules. 2023 May 5;28(9):3910. doi: 10.3390/molecules28093910 (PMC10180259; doi:10.3390/molecules28093910)
Supplement: Supplementary file 1 [file molecules-28-03910-s001.zip › molecules-2303104-supplementary.pdf]

SUPPLEMENTARY MATERIAL

Non-phenomenological description of the time-resolved emission in solution with quantum-classical vibronic approaches. Application to coumarin C153 in methanol

Javier Cerezo, Sheng Gao, Nicola Armaroli, Francesca Ingrosso, Giacomo Prampolini, Fabrizio Santoro, Barbara Ventura and Mariachiara Pastore

Contents

A QMD-FF parameterization **S3**

A.1 DFT training data S3

A.2 Parameterization procedure S6

B QMD-FF validation **S9**

B.1 QMD-FF potential energy surfaces S9

B.2 QMD-FF geometries S10

C QMD-FF parameters **S12**

D Molecular Dynamics details **S19**

E Steady state spectra **S20**

E.1 Gas phase spectra S20

E.2 Spectra in solution S24

F Transient spectra **S26**

F.1 Transient absorption analysis S26

F.2 Time-resolved Emission S27

F.3 Effect of the temperature of QM modes on TR spectra S28

F.4 Solvent Response S29

A QMD-FF parameterization

A.1 DFT training data

The ground state (S_0) optimized geometry found at PBE0/6-31G* level and shown in Figure S1 indicates a marked difference in the structure of four 6-membered rings constituting C153 backbone. In fact, the cycle bearing the CF_3 group and the neighboring one (red shaded surface

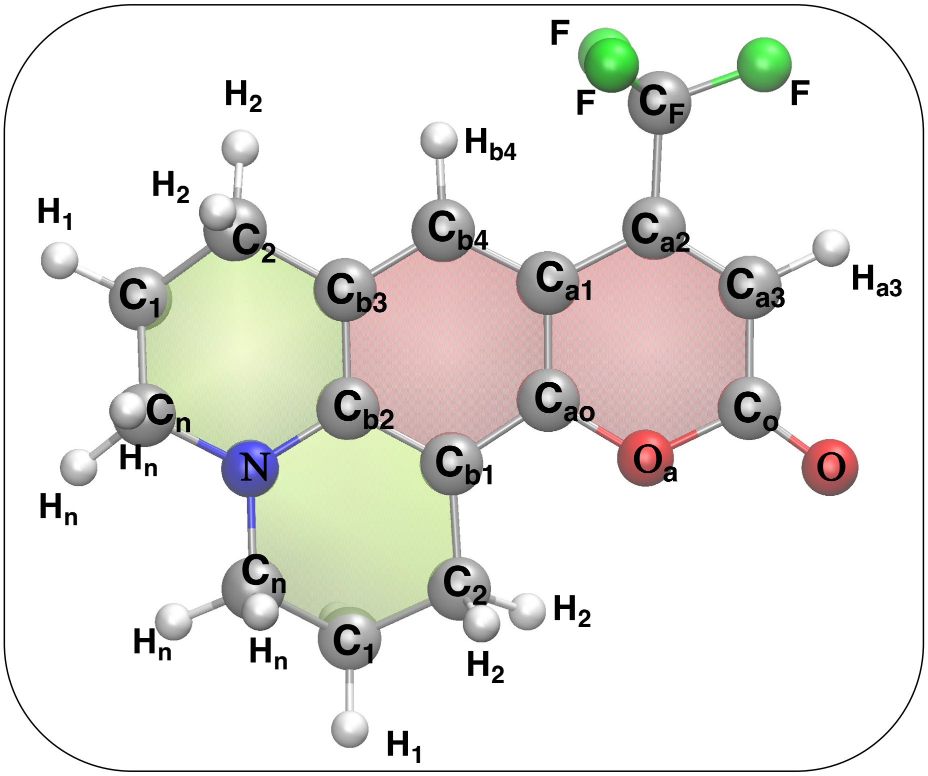

Figure S1: Optimized QM structure (PBE0/6-31G*) of coumarin C153 and atom types employed in QMD-FF parameterization of. both ground and electronic states. As in the main text (see Figure 1), red and green shaded surfaces indicate aromatic or aliphatic rings, respectively.

in Figure S1) show a "stiff" planar conformation, as could be expected from their aromatic character, whereas the two rings containing the N atoms (green surfaces, see also Figure 1.b in the main text) are not planar, being the C_1 atom significantly displaced out of the plane containing the rest of the carbon skeleton. Therefore, beside the flexible dihedral ruling the

rotation of the CF_3 group (δ_F), a certain degree of flexibility should be expected also for the non aromatic rings, leading to the definition of further "soft" coordinates as the dihedrals χ_1 and χ_2. In the optimized geometry, the latter are found at -48° and 48° , respectively, where the C_1 atoms are found below the backbone plane as shown in Figure S2.a and Figure 1.b in the main text, hence corresponding to a *sin* conformation. It should be however noticed that a rotation of one or both the χ dihedrals may displace the C_1 atoms either above or below the ring plane, thus more than one conformer should be expected.

The three possible *sin*, *anti*$_1$ (χ_1=χ_2=48°) and *anti*$_2$ (χ_1=χ_2=-48°) conformers are displayed in Figure S2. By looking at their relative energies ΔE, reported in Table S1, it is evident, on the

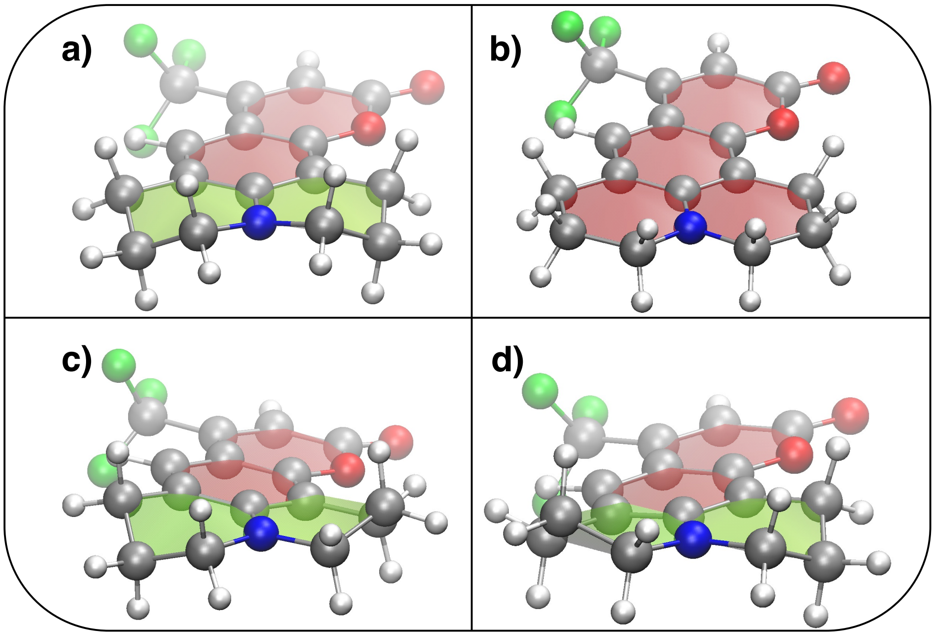

Figure S2: Investigated *sin*, *anti* and planar conformers of the C153 coumarin dye.

one hand, that only slight differences (∼ 1 kJ/mol) are found between the *sin* and the two degenerate *anti* conformers. On the other hand, it can be hypothesized that the transition between two conformers takes place through a planarization (χ_1=χ_2=0°) of the aliphatic rings. It can be hence interesting to evaluate the stability of a completely planar conformer, whose structure is displayed in Figure S2. Based on the results reported in Table S1, although the planar structure lies at a significantly higher energy with respect to the *syn* conformer, the two *anti* structures

Conformer	ΔE (kJ/mol)	VE (eV)	Osc. Str.
sin	0.0	3.50	0.36
$anti_1$	1.2	3.49	0.37
$anti_2$	1.2	3.49	0.37
planar	76.1	3.46	0.37

Table S1: Conformational energy (ΔE), first excited state vertical energy (VE) and oscillator strength for the different conformers of C153 displayed in Figure S2

can be instead expected to be populated at room temperature. Is might be therefore interesting to ascertain if the structural differences among the considered conformers affect the electronic transition properties. To this end, the vertical excitation energy (VE) and the oscillator strength to the first excited state is also reported for all conformations in Table S1 : it is evident that the conformational differences among the considered geometries only induce negligible effects on both the transition energy and its intensity. Indeed, only the planar conformer, which has a very low probability of being populated, is found very slightly red shifted (0.04 eV) with respect to the minimum *sin* conformer.

In conclusion, for all the above reasons, the QMD-FF parameterization for the ground state was carried out over a QM database containing the PBE0 optimized geometry and all its Hessian matrix elements computed at the minimized *syn* structure, and relaxed torsional profiles computed at the same level of theory along the δ_F and χ dihedrals. Finally, as far as the S_1 excited state FF is concerned, the same data were obtained at PBE0 level, yet resorting to the TD-DFT approach.

A.2 Parameterization procedure

The QMD-FF for a solvated system composed by C153 and a number (N_{MeOH}) of methanol molecules is partitioned in an inter-molecular term and an intra-molecular one:

$$E^{tot}_{QMD-FF} = E^{inter}_{QMD-FF} + E^{intra}_{QMD-FF} \tag{S1}$$

E^{inter}_{QMD-FF} describes the solute's interaction with the solvent and the interactions among solvent molecules, wheres E^{intra}_{QMD-FF} drives solute and solvent 's flexibility, i.e.

$$E^{intra}_{QMD-FF} = E^{intra_{C153}}_{QMD-FF} + E^{intra_{MeOH}}_{QMD-FF} \tag{S2}$$

The interactions between the solute and the solvent and among the solvent molecules are accounted for by the first term of equation (S1), that is

$$E^{inter}_{QMD-FF} = \sum_{i=1}^{N_{C153}} \sum_{j=1}^{N_{MeOH}} E^{C153-MeOH}_{ij} + \sum_{i=1}^{N_{MeOH}} \sum_{j=1}^{N_{MeOH}} E^{MeOH-MeOH}_{ij} \tag{S3}$$

where N_{C153} and N_{MeOH} are the number of coumarin and solvent atoms, respectively, and E^{x-MeOH}_{ij} (with x= C153, MeOH) is computed as the standard sum of a 12-6 LJ potential and Coulomb charge-charge interactions:

$$E^{x-MeOH}_{ij} = \left(4\varepsilon^{x-MeOH}_{ij}\left[\left(\frac{\sigma^{x-MeOH}_{ij}}{r_{ij}}\right)^{12} - \left(\frac{\sigma^{x-MeOH}_{ij}}{r_{ij}}\right)^{6}\right]\right) + \left(\frac{q^x_i q^{MeOH}_j}{(4\pi\varepsilon_0)r_{ij}}\right) \tag{S4}$$

All the parameters describing methanol were transferred from the Optimized Potential for Liquid Simulation (OPLS) database.[1,2] Conversely, the FF terms concerning with C153, that is $E^{C153-MeOH}_{ij}$ and $E^{intra_{C153}}_{QMD-FF}$, were refined specifically for the target system. The former intermolecular term was refined by deriving the solute point charges q^{C153}_i entering in eq. (S4) from QM calculations, purposely carried out on the optimized *syn* conformation of the isolated C153 coumarin. Concretely, all C153 atomic charges were derived from the PBE0/6-31G* electronic density computed at DFT (for the gropund state) or TD-DFT (for S_1) level through the CM5[3] procedure, accounting for the methanol solvent by means of the C-PCM[4] method. Consistently with the description adopted for the solvent, the LJ terms entering eq. (S4) were transferred for both states from the OPLS FF.[1,2]

The intramolecular term $E^{intra_{C153}}_{QMD-FF}$ was derived from the QM database described in section A through the JOYCE protocol. Notwithstanding all the details of JOYCE procedure can be found in the original papers,[5–7] a brief description of the specific parameterization of the C153 QMD-FF is given in the following. The intramolecular QMD-FF terms for the ground and excited state (S_1) of coumarin C153 both take the standard expression:

$$E^{intra(\mathbf{r}^{ric},\mathbf{R}^{ric})}_{QMD-FF} = E_s(\mathbf{r}^{ric}) + E_b(\mathbf{r}^{ric}) + E_{st}(\mathbf{r}^{ric}) + E_{ft}(\mathbf{R}^{ric}) + E^{intra}_{Nb}(\mathbf{R}^{ric}) \tag{S5}$$

where the first three terms refer to stretching (E_s), angle bending (E_b) and small dihedral (E_{st}) distortions, depend on stiff redundant internal coordinates (RICs), $\mathbf{r}^{ric}$, and are therefore approximated through harmonic potentials:

$$E_s = \frac{1}{2}\sum_i^{N_{bonds}} k_i^s(r_i - r_i^0)^2 \ ; \ E_b = \frac{1}{2}\sum_i^{N_{angles}} k_i^b(\theta_i - \theta_i^0)^2 \ ; \ E_{st} = \frac{1}{2}\sum_i^{N_{dihedrals}} k_i^{st}(\phi_i - \phi_i^0)^2 \tag{S6}$$

E_{ft} depends on the dihedrals δ_F and χ, which are expected to present an enhanced flexibility,[8] (soft RICs, $\mathbf{R}^{ric}$), and are hence represented by Fourier-like expansions

$$E_{ft} = \sum_{\mu}^{N_{dihedrals}} \sum_{j}^{N_{cos\mu}} k^{ft}_{j\mu}\left[1 + cos(n^{\mu}_j \delta_\mu - \gamma^{\mu}_j)\right] \tag{S7}$$

Finally, the last term of equation (S5) also contributes to the energy of the χ dihedrals and again depends on a soft RIC, namely the intra-molecular distance between C_1 coumarin atoms (see Figure S1 for definition).

$$E^{intra}_{Nb} = \sum_i \sum_{i<j} 4\varepsilon^{intra}_{ij}\left[\left(\frac{\sigma^{intra}_{ij}}{r_{ij}}\right)^{12} - \left(\frac{\sigma^{intra}_{ij}}{r_{ij}}\right)^{6}\right] + \left(\frac{q^{intra}_i q^{intra}_j}{(4\pi\varepsilon_0)r_{ij}}\right) \tag{S8}$$

It is worth mentioning that according to the JOYCE protocol[5] intra-molecular LJ parameters are allowed to be different from the ones employed for the description of inter-molecular interactions in equation S4, and they can be included in the QMD-FF just for selected intra-molecular atom pairs, thus allowing for a more specific parameterization.

The parameters defining the torsional potential term governing the rotation of the χ dihedrals and those entering eq. (S8) were fitted directly on the DFT and TD-DFT relaxed torsional profiles and thereafter constrained throughout the successive parameterizations. All other intramolecular QMD-FF parameters were obtained with the JOYCE code,[9] by performing the

usual two-step procedure: a first cycle which fits all harmonic parameters at once and a second cycle, in which the harmonic parameters are fixed and the parameters for the remaining flexible dihedral δ_F are parameterized against the QM scans. All final parameters are reported in the next section in Tables S2 to S12.

B QMD-FF validation

B.1 QMD-FF potential energy surfaces

Several validation tests were carried out on both the ground-state (S_0) and excited-state (S_1) QMD-FFs and selected results are shown in Figure S3. In panel a) the overlap between the structures optimized at QM level with thos obtained by minimizing the QMD-FF are shown for both states, confirming the high accuracy of the QMD-FF in reproducing the minimum energy structures in both S_0 and S_1. The QMD-FF reliability in mimicking the small oscillations

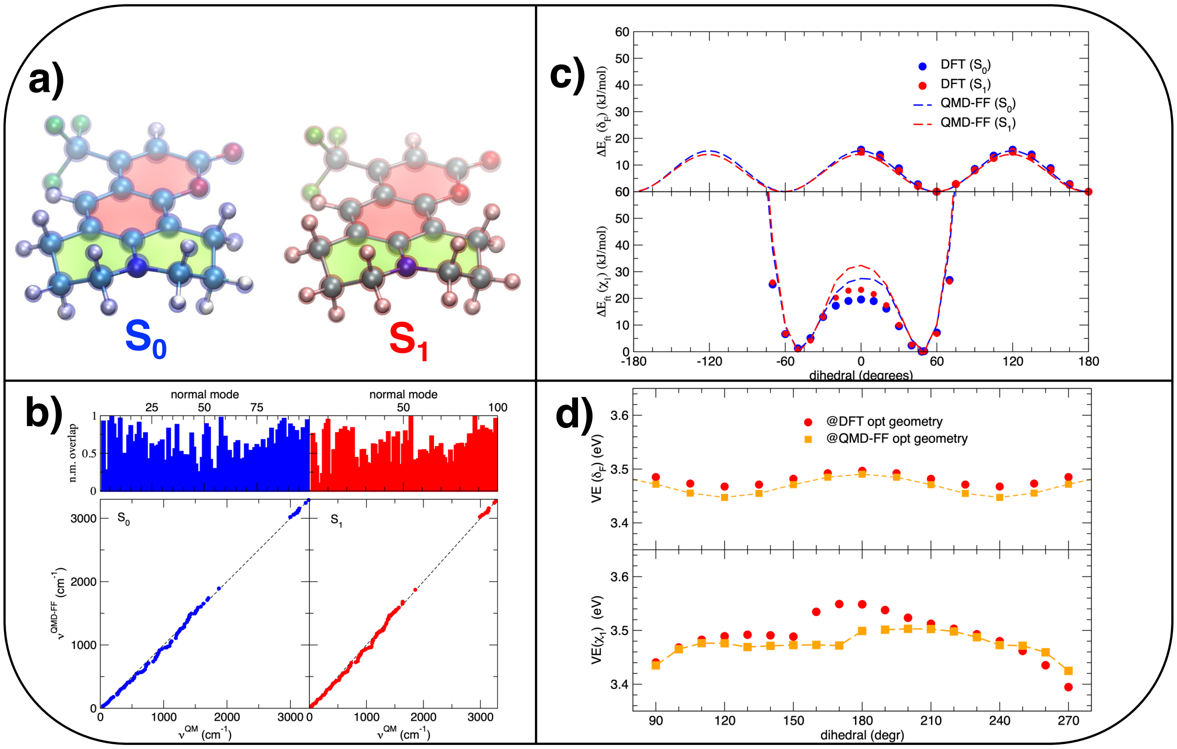

Figure S3: Validation tests summary: a) overlap of the structures optimized at QM (DFT, full spheres) and QMD-FF (transparent blueish or reddish spheres) level, obtained for the ground and the excited state; b) comparison of QM and QMD-FF vibrational frequencies (bottom panel) and normal mode overlap (top panel); c)relaxed torsional energy profiles (ΔE_{ft}), obtained for δ_F (top) and χ_1 dihedrals (bottom) at QM (DFT, symbols) and QMD-FF (dashed lines) level, for the ground (blue) and excited (red) states; d) vertical energies (VE) computed, at TD-DFT level, as a function of the flexible dihedrals in the QM (red circles) and QMD-FF (orange circles) optimized geometries obtained along the relaxed scans shown in panel c).

around the dye's equilibrium structure is instead confirmed in Figure S3.b, where the vibrational

frequencies and the overlap between the corresponding normal modes are compared for both states at QM and QMD-FF level. The DFT and FF descriptions can be also compared over larger portions of the dye's PES, as those spanned by the rotation of the flexible dihedrals δ_F, χ_1 and χ_2 defined in Figure 1 in the main text. Figure S3.c compares, for the two considered electronic states, the relaxed torsional energy profiles of δ_F and χ_1 obtained by constrained optimization using either DFT or the QMD-FF. Similar results were obtained when considering χ_1, which is not shown for the sake of clarity.

B.2 QMD-FF geometries

Finally, to validate their adoption in the present study, the two C153 QMD-FFs were eventually tested in their capability to deliver reliable and accurate structures, which could be directly employed in the QM calculations required by the CEA-VE or Ad-MD$|g$VH protocols without any further refinement. To this end, we computed the vertical excitation to the first excited state for all the relaxed structures obtained in the scans carried out either at QM or at FF level and shown in Figure S3.c. The results of this first test are summarized in panel d) of the same Figure. The agreement is very good, except, for the χ_2 profile, in the region where the external ring approaches planarity. Similar results (not shown) were obtained for χ_2. However, considering the high barrier at ($\chi_2 \sim 0^\circ$) such structure should be seldom populated at room temperature, hence their impact on the final spectra should be negligible. Indeed, from a first visual inspection of the MD runs carried out *in vacuo* at 383 K, did not reveal any nonphysical distortions, and C153 molecules appears to vibrate around the correct (QM) minimum structure in both states. For a deeper insight, as displayed in Figure S4, the population distributions of the dihedrals achieved in the MD runs can be compared. The distribution confirms what expected from the torsional profiles: the δ_F, due to the smaller barrier heights, populates several conformers, while, in both states, C153 remains in the initial *sin* conformation, since the χ barrier at 0° can't be crossed even at 383 K.

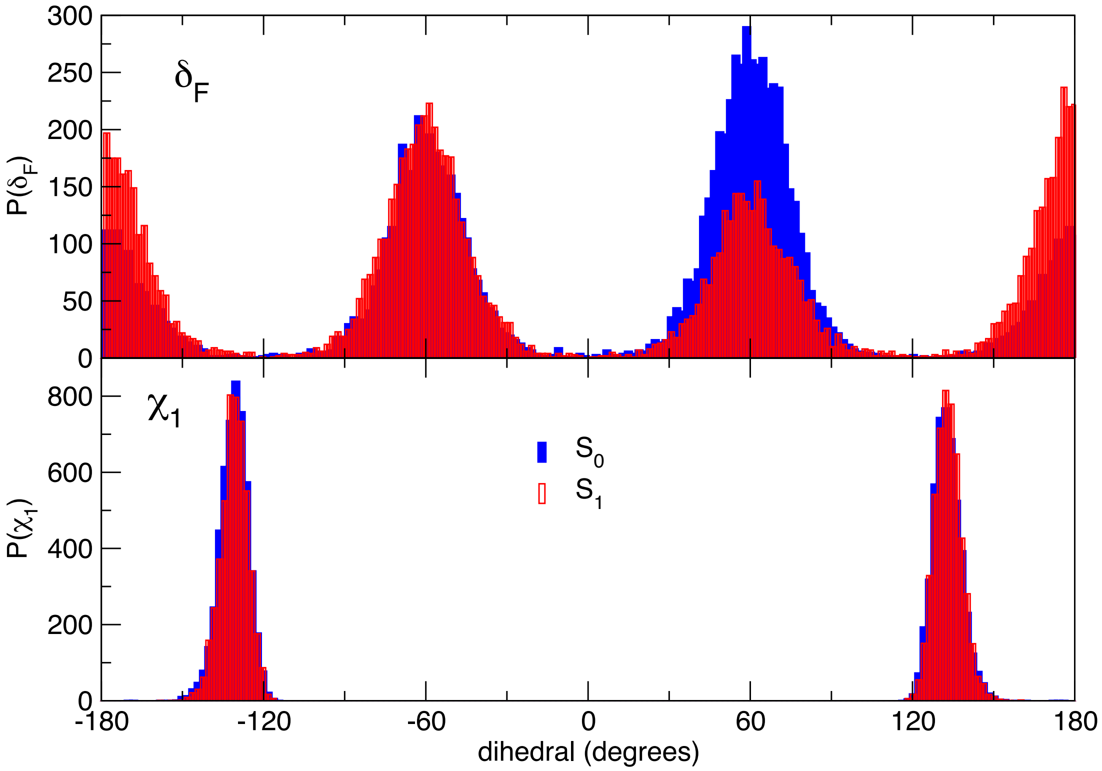

Figure S4: Population distribution for the most flexible dihedrals achieved during the simulations performed in vacuo at 383 K with the ground (blue) and excited (red) state QMD-FF, respectively.

C QMD-FF parameters

Atom type	σ	ε	q^{S_0}	q^{S_1}
O_A	2.900	0.5858	-0.1558	-0.1857
C_{AO}	3.550	0.2929	0.0977	0.0935
C_{A1}	3.550	0.2929	-0.0508	-0.0184
C_{A2}	3.550	0.2929	-0.0054	-0.0778
C_{A3}	3.550	0.2929	-0.1118	-0.1320
C_O	3.750	0.4393	0.2450	0.1853
C_{B1}	3.550	0.2929	-0.0422	0.0087
C_{B2}	3.550	0.2929	0.1155	0.1184
C_{B3}	3.550	0.2929	-0.0307	-0.0272
C_{B4}	3.550	0.2929	-0.1005	-0.1034
N	3.250	0.7113	-0.3113	-0.2352
C_1	3.500	0.2761	-0.1563	-0.1492
C_2	3.500	0.2761	-0.1553	-0.1486
C_N	3.500	0.2761	-0.0472	-0.0339
O	2.960	0.8786	-0.3606	-0.4068
C_F	3.500	0.2761	0.3417	0.3179
F	2.950	0.2218	-0.1223	-0.1465
H_{A3}	2.420	0.1255	0.1079	0.1073
H_{B4}	2.420	0.1255	0.1231	0.1099
H_1	2.500	0.1255	0.0952	0.1007
H_2	2.500	0.1255	0.1017	0.1065
H_N	2.500	0.1255	0.1131	0.1267

Table S2: QMD-FF intermolecular LJ parameters (σ,Å) and ε, kJ/mol) and point charges for the ground (q^{S_0}) and for the excited state (q^{S_1}) of coumarin C153. Atom types are shown in Figure S1

**stretching**	**r^0**	**k^s stretching**	**r^0**	**k^s**	
O_{A}-C_{AO}	1.361	3019.11	C_{AO}-C_{A1}	1.405	2550.15
C_{A1}-C_{A2}	1.436	2902.07	C_{A2}-C_{A3}	1.354	4668.16
C_{A3}-C_{O}	1.450	2299.55	O_{A}-C_{O}	1.382	2071.16
C_{AO}-C_{B1}	1.391	3247.05	C_{B1}-C_{B2}	1.412	2752.30
C_{B2}-C_{B3}	1.425	2510.09	C_{B3}-C_{B4}	1.378	3481.73
C_{A1}-C_{B4}	1.405	3153.40	C_{B2}-N	1.377	3360.95
N-C_{N}	1.449	2405.79	C_{N}-C_{1}	1.518	2245.36
C_{1}-C_{2}	1.521	2350.55	C_{B1}-C_{2}	1.506	2404.14
C_{B3}-C_{2}	1.508	2453.60	C_{O}-O	1.205	7413.46
C_{A2}-C_{F}	1.508	2546.39	C_{F}-F	1.344	2714.42
C_{B4}-H_{A3}	1.085	3445.30	C_{A3}-H_{B4}	1.082	3542.97
C_{N}-H_{N}	1.104	3059.47	C_{1}-H_{1}	1.097	3187.82

Table S3: Intramolecular stretching parameters for the ground-state of C153. Equilibrium distances r^0 are in Å and force constants k^s in kJ/mol Å^{-2}.

**bending**	θ^0	k^b	**bending**	θ^0	k^b
C_{AO}-O_A-C_O	123.4	548.82	O_A-C_{AO}-C_{A1}	121.2	820.74
C_{A1}-C_{AO}-C_{B1}	123.1	146.62	O_A-C_{AO}-C_{B1}	115.7	970.24
C_{AO}-C_{A1}-C_{A2}	117.0	375.71	C_{AO}-C_{A1}-C_{B4}	117.0	455.98
C_{A2}-C_{A1}-C_{B4}	126.0	868.25	C_{A1}-C_{A2}-C_{A3}	121.0	619.54
C_{A3}-C_{A2}-C_F	119.3	639.30	C_{A1}-C_{A2}-C_F	119.7	220.74
C_{A2}-C_{A3}-C_O	121.5	613.01	O_A-C_O-C_{A3}	116.0	336.36
O_A-C_O-O	118.0	956.71	C_{A3}-C_O-O	126.0	171.95
C_{AO}-C_{B1}-C_{B2}	118.5	449.50	C_{AO}-C_{B1}-C_2	119.8	590.87
C_{B2}-C_{B1}-C_2	121.8	718.50	C_{B1}-C_{B2}-C_{B3}	119.6	360.28
C_{B1}-C_{B2}-N	120.1	779.98	C_{B3}-C_{B2}-N	120.3	614.36
C_{B2}-C_{B3}-C_{B4}	119.6	547.96	C_{B2}-C_{B3}-C_2	120.3	703.07
C_{B4}-C_{B3}-C_2	120.1	670.08	C_{A1}-C_{B4}-C_{B3}	122.3	470.57
C_{B2}-N-C_N	119.8	539.72	C_N-N-C_N	116.3	796.43
N-C_N-C_1	111.1	577.06	C_N-C_1-C_2	109.3	873.92
C_{B3}-C_2-C_1	111.1	332.78	C_{B1}-C_2-C_1	111.0	414.94
C_{A2}-C_F-F	111.1	596.68	F-C_F-F	107.4	867.28
C_{AO}-C_{A3}-H_{B4}	176.7	449.91	C_O-C_{A3}-H_{B4}	116.2	220.25
C_{B3}-C_{B4}-H_{A3}	118.3	286.39	C_{A1}-C_{B4}-H_{A3}	119.4	397.65
N-C_N-H_N	111.0	480.66	C_1-C_N-H_N	109.9	386.62
C_N-C_1-H_1	109.6	383.08	C_2-C_1-H_1	111.0	387.61
C_{B1}-C_2-H_2	109.7	416.03	C_{B3}-C_2-H_2	109.7	408.75
C_1-C_2-H_2	109.0	397.37	H_N-C_N-H_N	107.0	358.77
H_1-C_1-H_1	107.5	347.21	H_2-C_2-H_2	105.7	352.83

Table S4: Intramolecular bending parameters for the ground-state of C153. Equilibrium angles θ^0 are in degree force constants k^b in kJ/mol rad^{-2}.

**dihedral**	ϕ^0	$\mathbf{k^t}$	**dihedral**	ϕ^0	$\mathbf{k^t}$
O_A-C_{AO}-C_{A1}-C_{A2}	0.0	39.77	C_{AO}-C_{A1}-C_{A2}-C_{A3}	0.0	39.77
C_{A1}-C_{A2}-C_{A3}-C_O	0.0	39.77	C_{A2}-C_{A3}-C_O -O_A	0.0	39.77
C_{AO}-O_A-C_O-C_{A3}	0.0	39.77	C_O -O_A -C_{AO}-C_{A1}	0.0	39.77
C_{A1}-C_{AO}-C_{B1}-C_{B2}	0.0	39.77	C_{B1}-C_{AO}-C_{A1}-C_{B4}	0.0	39.77
C_{AO}-C_{A1}-C_{B4}-C_{B3}	0.0	39.77	C_{B2}-C_{B3}-C_{B4}-C_{A1}	0.0	39.77
C_{B1}-C_{B2}-C_{B3}-C_{B4}	0.0	39.77	C_{AO}-C_{B1}-C_{B2}-C_{B3}	0.0	39.77
O_A-C_{AO}-C_{A1}-C_{B4}	180.0	39.77	C_{B4}-C_{A1}-C_{A2}-C_{A3}	180.0	39.77
C_O-O_A-C_{AO}-C_{B1}	180.0	39.77	C_{B1}-C_{AO}-C_{A1}-C_{A2}	180.0	39.77
O_A-C_{AO}-C_{B1}-C_{B2}	180.0	39.77	C_{A2}-C_{A1}-C_{B4}-C_{B3}	180.0	39.77
O_A-C_{AO}-C_{B1}-C_2	0.0	227.51	C_2 -C_{B1}-C_{B2}-N	0.0	159.17
N-C_{B2}-C_{B3}-C_2	0.0	230.82	C_{B4}-C_{A1}-C_{A2}-C_F	0.0	42.20
C_{A2}-C_{A1}-C_{A3}-C_F	0.0	39.77	C_{AO}-C_{B2}-C_2 -C_{B1}	0.0	39.77
C_{B4}-C_{B2}-C_2-C_{B3}	0.0	39.77	C_{A1}-C_{AO}-C_{B4}-N	180.0	39.77
C_{A3}-O_A-C_O-O	180.0	283.09	C_{B4}-C_{A1}-C_{B3}-H_{A3}	0.0	540.19

Table S5: Intramolecular parameters for stiff torsions for the ground-state of C153. Equilibrium dihedral angles ϕ^0 are in degrees and force constants k^t in kJ/mol rad^{-2}.

**dihedral**	N_{cos}	n	k^d (kJ/mol)	γ (degr)
C_{A1}-C_{A2}-C_F-F	2	0	-0.122	0.00
(δ_F)		3	2.556	0.00
C_1-C_N-C_2-C_{B2}	7	0	88.577	0.00
($\chi_{1(2)}$)		1	535.366	0.00
		2	-187.368	180.00
		3	-40.315	0.00
		4	-66.781	0.00
		5	-37.796	0.00
		6	-12.028	0.00

Table S6: Flexible dihedrals parameters for C153 ground-state QMD-FF: number of cosines n, γ (degrees) and force constants k^d in kJ/mol.

Pair (ij)	σ_{ij}^{intra}	ε_{ij}^{intra}
C_1-C_1	3.500	0.2761

Table S7: QMD-FF parameters for non-bonded intramolecular interactions in the ground state: σ^{intra} (Å) and ε^{intra} (kJ/mol).

**stretching**	$\mathbf{r^0}$	$\mathbf{k^s}$ **stretching**	$\mathbf{r^0}$	$\mathbf{k^s}$	
O_A-C_{AO}	1.343	3326.55	C_{AO}-C_{A1}	1.393	2867.03
C_{A1}-C_{A2}	1.476	1862.10	C_{A2}-C_{A3}	1.384	4147.39
C_{A3}-C_O	1.421	3144.38	O_A-C_O	1.435	1453.85
C_{AO}-C_{B1}	1.413	2388.46	C_{B1}-C_{B2}	1.425	2552.22
C_{B2}-C_{B3}	1.412	2648.15	C_{B3}-C_{B4}	1.387	3478.22
C_{A1}-C_{B4}	1.407	2974.23	C_{B2}-N	1.383	2737.91
N-C_N	1.448	2201.56	C_N-C_1	1.520	2288.19
C_1-C_2	1.522	2315.02	C_{B1}-C_2	1.495	2076.50
C_{B3}-C_2	1.505	2234.40	C_O-O	1.215	6864.93
C_{A2}-C_F	1.469	2469.33	C_F-F	1.364	2289.65
C_{B4}-H_{A3}	1.085	3447.94	C_{A3}-H_{B4}	1.084	3502.52
C_N-H_N	1.105	3068.59	C_1-H_1	1.097	3195.65

Table S8: QMD-FF intramolecular stretching parameters for for C153 first excited state (S_1). Equilibrium distances r^0 are in Å and force constants k^s in kJ/mol Å^{-2}.

bending	θ^0	k^b	bending	θ^0	k^b
C_{AO}-O_A-C_O	121.5	671.29	O_A-C_{AO}-C_{A1}	124.0	421.43
C_{A1}-C_{AO}-C_{B1}	121.6	316.51	O_A-C_{AO}-C_{B1}	114.5	569.74
C_{AO}-C_{A1}-C_{A2}	117.2	701.92	C_{AO}-C_{A1}-C_{B4}	117.6	419.45
C_{A2}-C_{A1}-C_{B4}	125.3	780.03	C_{A1}-C_{A2}-C_{A3}	117.5	516.45
C_{A3}-C_{A2}-C_F	122.3	606.62	C_{A1}-C_{A2}-C_F	120.2	164.70
C_{A2}-C_{A3}-C_O	124.4	447.85	O_A-C_O-C_{A3}	115.5	288.72
O_A-C_O-O	115.3	993.46	C_{A3}-C_O-O	129.2	167.77
C_{AO}-C_{B1}-C_{B2}	118.9	538.58	C_{AO}-C_{B1}-C_2	119.2	683.89
C_{B2}-C_{B1}-C_2	121.9	476.05	C_{B1}-C_{B2}-C_{B3}	120.2	380.17
C_{B1}-C_{B2}-N	118.8	467.10	C_{B3}-C_{B2}-N	121.0	404.96
C_{B2}-C_{B3}-C_{B4}	118.2	591.46	C_{B2}-C_{B3}-C_2	120.7	448.55
C_{B4}-C_{B3}-C_2	121.1	570.67	C_{A1}-C_{B4}-C_{B3}	123.5	519.28
C_{B2}-N-C_N	121.0	536.06	C_N-N-C_N	117.3	694.54
N-C_N-C_1	111.7	609.88	C_N-C_1-C_2	109.7	839.36
C_{B3}-C_2-C_1	110.6	524.96	C_{B1}-C_2-C_1	111.6	518.89
C_{A2}-C_F-F	112.8	574.58	F-C_F-F	107.0	801.09
C_{AO}-C_{A3}-H_{B4}	176.7	397.86	C_O-C_{A3}-H_{B4}	115.0	219.76
C_{B3}-C_{B4}-H_{A3}	118.1	304.59	C_{A1}-C_{B4}-H_{A3}	118.4	388.32
N-C_N-H_N	109.3	482.88	C_1-C_N-H_N	109.9	368.23
C_N-C_1-H_1	108.9	397.08	C_2-C_1-H_1	111.3	396.91
C_{B1}-C_2-H_2	109.3	406.50	C_{B3}-C_2-H_2	110.3	421.60
C_1-C_2-H_2	109.4	391.45	H_N-C_N-H_N	107.1	355.49
H_1-C_1-H_1	107.5	344.98	H_2-C_2-H_2	105.0	344.42

Table S9: QMD-FF intramolecular bending parameters for C153 first excited state (S_1). Equilibrium angles θ^0 are in degree force constants k^b in kJ/mol rad^{-2}.

**dihedral**	ϕ^0	**k^t**	**dihedral**	ϕ^0	**k^t**
O_A-C_{AO}-C_{A1}-C_{A2}	0.0	40.00	C_{AO}-C_{A1}-C_{A2}-C_{A3}	0.0	40.00
C_{A1}-C_{A2}-C_{A3}-C_O	0.0	40.00	C_{A2}-C_{A3}-C_O -O_A	0.0	40.00
C_{AO}-O_A-C_O-C_{A3}	0.0	40.00	C_O -O_A -C_{AO}-C_{A1}	0.0	40.00
C_{A1}-C_{AO}-C_{B1}-C_{B2}	0.0	40.00	C_{B1}-C_{AO}-C_{A1}-C_{B4}	0.0	40.00
C_{AO}-C_{A1}-C_{B4}-C_{B3}	0.0	40.00	C_{B2}-C_{B3}-C_{B4}-C_{A1}	0.0	40.00
C_{B1}-C_{B2}-C_{B3}-C_{B4}	0.0	40.00	C_{AO}-C_{B1}-C_{B2}-C_{B3}	0.0	40.00
O_A-C_{AO}-C_{A1}-C_{B4}	180.0	40.00	C_{B4}-C_{A1}-C_{A2}-C_{A3}	180.0	40.00
C_O-O_A-C_{AO}-C_{B1}	180.0	40.00	C_{B1}-C_{AO}-C_{A1}-C_{A2}	180.0	40.00
O_A-C_{AO}-C_{B1}-C_{B2}	180.0	40.00	C_{A2}-C_{A1}-C_{B4}-C_{B3}	180.0	40.00
O_A-C_{AO}-C_{B1}-C_2	0.0	224.04	C_2 -C_{B1}-C_{B2}-N	0.0	146.16
N-C_{B2}-C_{B3}-C_2	0.0	224.99	C_{B4}-C_{A1}-C_{A2}-C_F	0.0	111.25
C_{A2}-C_{A1}-C_{A3}-C_F	0.0	40.00	C_{AO}-C_{B2}-C_2 -C_{B1}	0.0	40.00
C_{B4}-C_{B2}-C_2-C_{B3}	0.0	40.00	C_{A1}-C_{AO}-C_{B4}-N	180.0	40.00
C_{A3}-O_A-C_O-O	180.0	212.45	C_{B4}-C_{A1}-C_{B3}-H_{A3}	0.0	504.20

Table S10: QMD-FF intramolecular parameters for stiff torsions for for C153 first excited state (S_1). Equilibrium dihedral angles ϕ^0 are in degrees and force constants k^t in kJ/mol rad^{-2}.

**dihedral**	N_{cos}	n	k^d (kJ/mol)	γ (degr)
C_{A1}-C_{A2}-C_F-F	2	0	-0.129	0.00
		3	2.338	0.00
C_1-C_N-C_2-C_{B2}	7	0	87.879	0.00
		1	532.429	0.00
		2	-185.656	180.00
		3	-41.954	0.00
		4	-65.223	0.00
		5	-36.589	0.00
		6	-10.986	0.00

Table S11: Intramolecular parameters for flexible torsions of the QMD-FF of coumarin's first excited state: number of cosines n, γ(degrees) and force constants k^d in kJ/mol.

Pair (ij)	σ_{ij}^{intra}	ε_{ij}^{intra}
C_1-C_1	3.500	0.2761

Table S12: QMD-FF parameters for non-bonded intramolecular interactions in C153 S_1 state: σ^{intra} (Å) and ε^{intra} (kJ/mol).

D Molecular Dynamics details

In all MD runs, no bond length was constrained and the time step was hence set to 0.25 fs. Temperature (T, 383 K and 298 K, for gas phase and solution, respectively) and pressure (P, 1 atm) were kept constant through the velocity-rescale[10] and Parrinello-Rahman[11] schemes, with coupling constant τ_T and τ_P set to 0.1 ps and 5 ps, respectively. A cut-off distance of 12 Å was employed for short-range interactions, and the standard correction for energy and virial applied to LJ potentials. Long range electrostatic was instead accounted for by the particle mesh Ewald scheme (PME). As far as equilibrium MD is concerned, in the gas phase, a first equilibration of 5 ns on the isolated C153 dye was followed by a production run of 10 ns in the NVT ensemble. In solution, all production runs lasted again 10 ns, yet in the NPT ensemble, where the system was previously equilibrated for 5 ns. In all cases, one hundred frames (one each 100 ps) containing the C153 dyes and a number of selected methanol molecules were extracted along each MD trajectory, according to the schemes discussed in the main text.

To follow the solvent response to the dye's electronic excitation, non equilibrium MD runs were prepared by extracting 500 starting configurations from the NPT production run carried out with the S_0 QMD-FF, simulating for each frame the system evolution after chenging the FF to S_1 for 100 ps, storing the resulting trajectories each 10 fs.

E Steady state spectra

E.1 Gas phase spectra

Before addressing the analysis of the spectral shapes in solution, a preliminary validation of our computational procedures was carried out in the gas phase. In fact, focusing on the isolated chromophore allows for a more direct and balanced comparison among the three different approaches discussed in this work, namely, the static vibronic FC|VH protocol, and the two schemes based on MD sampling, CEA-VE and Ad-MD|gVH. It is here worth stressing that notwithstanding FC|VH treats all vibrations at quantum level, it is based on harmonic expansions of the initial and final PES at the ground-state equilibrium geometry. CEA-VE is instead based on a dynamical sampling of all vibrations which move classically, yet according to an anharmonic QMD-FF. Finally, the recently proposed Ad-MD|gVH approach is still based on the same MD adopted in the CEA-VE calculation, but it treats at classical level only the soft motions of the molecule, whilst, for each sampled frame along the soft mode dynamics, all stiff modes are accounted for at quantum level. Before applying the latter strategy to condensed phase simulations, it is therefore of key importance to assess a rigorous partition into dye's soft and stiff modes. As evidenced in Figure 2.b in the main text, chemical intuition suggests that C153 intramolecular soft-modes are connected to the rotation of the CF_3 group (δ_F) and, possibly, to ring distortions around the N and O_a ring atoms (see Figure S1). Whereas the first rotation is easily defined as a combination of three dihedral angles, the latter motions have a more elusive nature. The most straightforward way to remove them from the set of the stiff modes is to project out all the 8 dihedral angles involving N or O_a. With this choice we shift in total 9 degrees of freedom to the classical set. It might be worth mentioning that, beside chemical intuition, the classical/quantum partition can be evaluated *a posteriori*, by checking the fraction of snapshots for which the reduced-dimensionality harmonic model in the stiff modes bears no imaginary frequencies, and is therefore suitable for a vibronic computation. With the present partition, this fraction is larger than 95% providing an adequate statistical set for the computation.[8]

Figure S5 compares the outcomes of the different computational schemes employed with

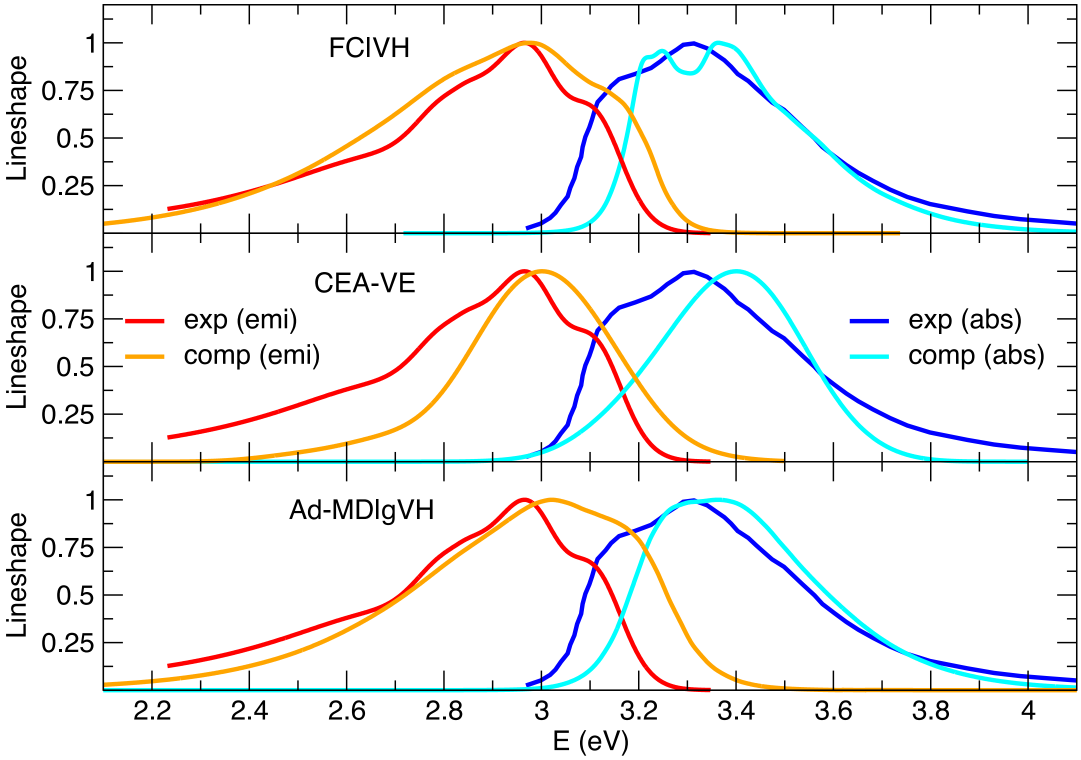

Figure S5: Absorption (abs) and emission (emi) spectra of C153 in the gas phase at 383 K, experimentally measured (blue and red lines) or computed by different methods: static (top panel), CEA-VE (middle panel) and Ad-MD|*g*VH (bottom panel). In all panels lineshape is reported in arbitrary units. For the static protocol, all spectra are computed at the FC|VH level.

absorption and emission steady-state lineshapes measured at 383 K.[12] By looking at the top panel, it appears that the fully quantum approach (FC|VH) provides spectra in very good agreement with experiment, both as far as the position and the shape are concerned. A similar result was already reported in ref.[13] by some of us, adopting the alternative FC|AH model. Here, since we plan to compute spectra in solution through our MQC protocol, we repeated the calculation with the FC|VH scheme, on which the Ad-MD|*g*VH approach is based. Figure S6 shows that FC|VH and FC|AH deliver, for C153, very similar results.

Turning to the MD based schemes, CEA-VE calculations are displayed in the middle panel of Figure S5 and perform similarly as far as the position of the spectra is concerned (apart from a slightly more-accentuated blue shift of the absorption). On the contrary, the agreement

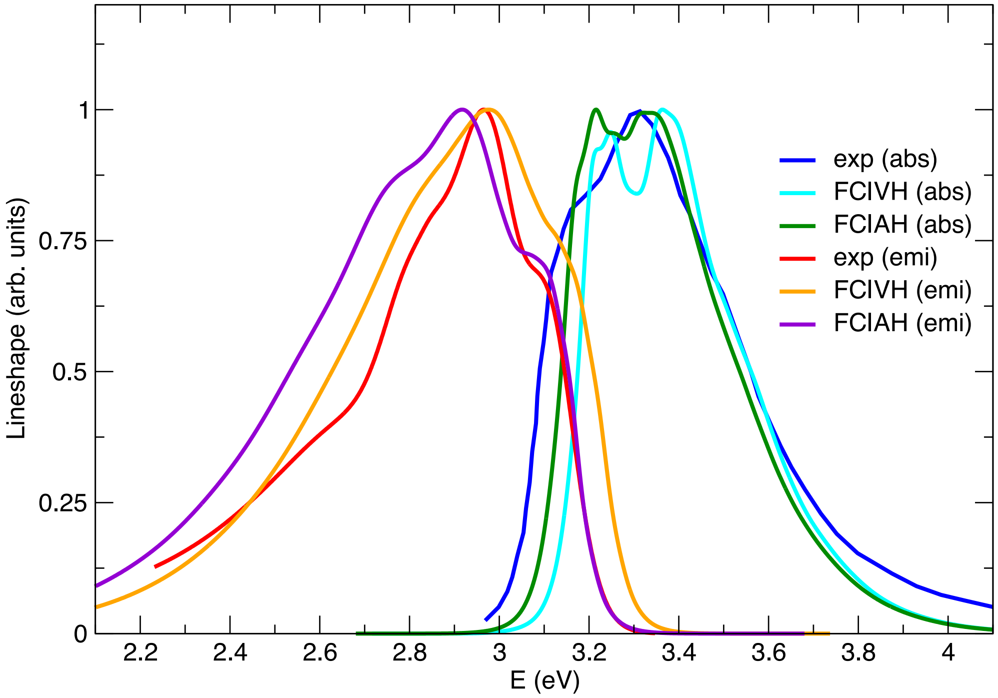

Figure S6: Absorption (abs) and emission (emi) spectra of C153 in the gas phase at 383 K, experimentally measured (blue and red lines) or computed by different methods according to the static approach: FC|VH (cyan and orange lines for abs and emi, respectively) and FC|AH (green and violet lines).

on the shape of the spectra clearly deteriorates. Beside the loss of vibronic resolution, the general asymmetry of the spectra is not reproduced, being even opposite for the absorption, where the longer tail toward the blue is replaced by a tail toward the red. This finding is not surprising since, considering the spectrum like a distribution, its asymmetry is connected to moments higher than the second one, which by theory have values at quantum level that are not conserved in classical and semi-classical approximations.[14] The Ad-MD|gVH method, conversely, provides spectra of quality similar to the fully quantum FC|VH approach, apart from a moderate loss of vibronic resolution which, at least in absorption, was slightly too accentuated at FC|VH level, and is smoothed too much by Ad-MD|gVH. We attribute this behavior to the intricate nature of the ring distortions around the N and O ring atoms in C153. Moving to the classical set the nine degrees of freedom described previously, we mainly remove modes with small vibrational frequency (< 300 cm^{-1}), apart from a pair of them characterized by an higher

frequency. Such modes can contribute to vibronic progressions to the fully-quantum spectrum whereas they only provide a broadening when treated classically. On balance however, Ad-MD$|g$VH appears to be robust and yields spectral signal with good accuracy, prompting us to confidently employ the MQC approach for all calculations in solution.

E.2 Spectra in solution

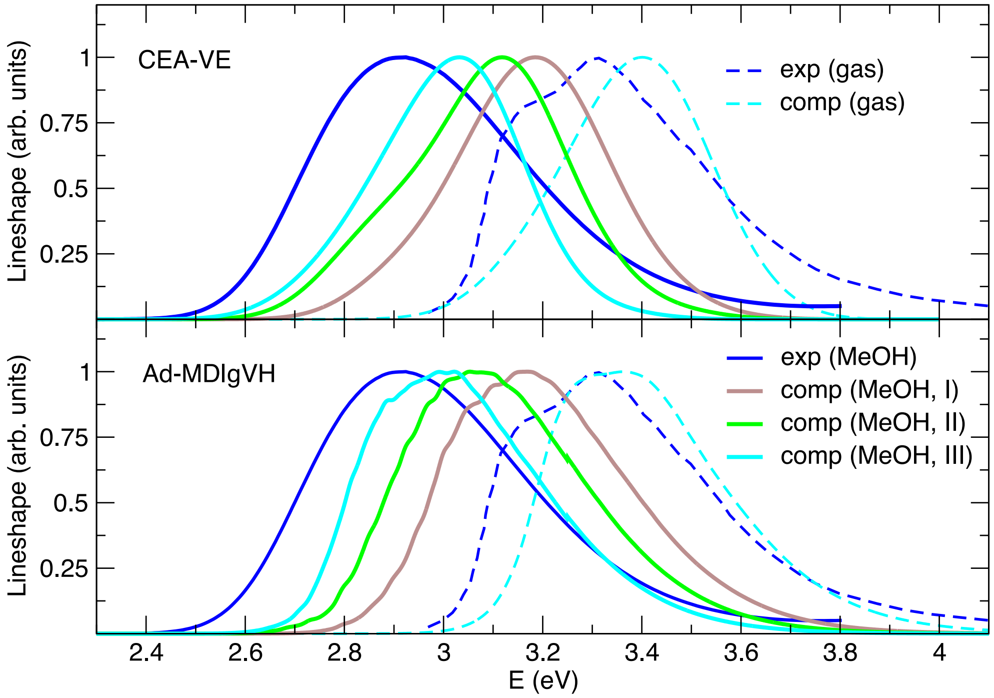

Figure S7: Absorption spectra of C153 in vacuo at 383 K (dashed lines) and in methanol solution (solid lines) at 300 K, experimentally measured (blue lines) or computed according to either CEA-VE (top panel) or Ad-MD|gVH (bottom panel), accounting for the solvent with the different schemes outlined in the main text.

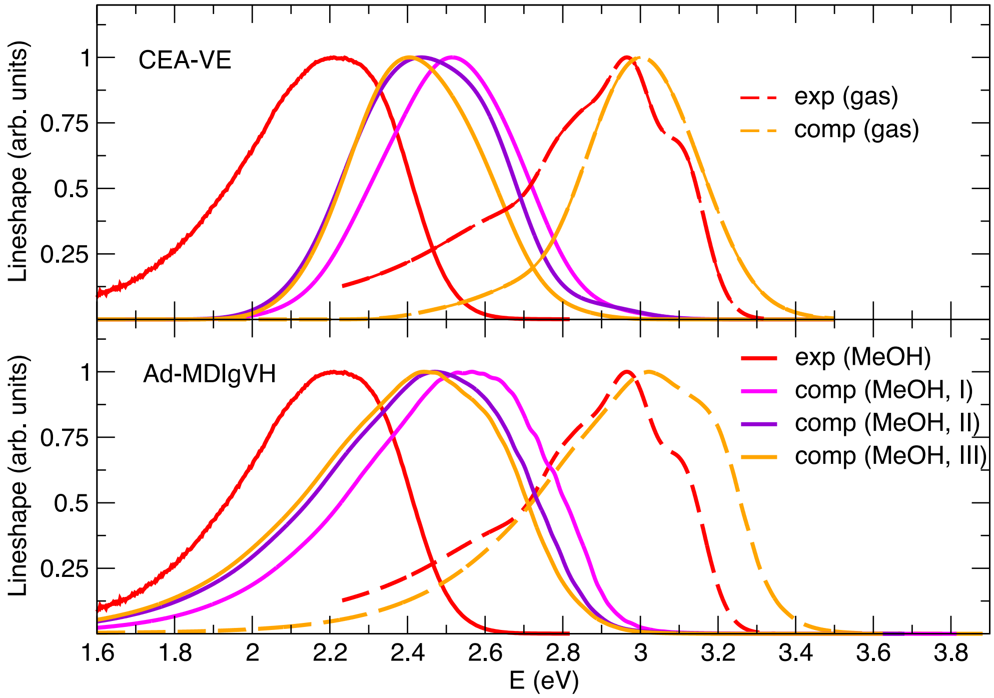

Figure S8: Emission spectra of C153 in vacuo at 383 K (dashed lines) and in methanol solution (solid lines) at 300 K, experimentally measured (red lines) or computed according to either CEA-VE (top panel) or Ad-MD|gVH (bottom panel), accounting for the solvent with the different schemes outlined in the main text.

F Transient spectra

F.1 Transient absorption analysis

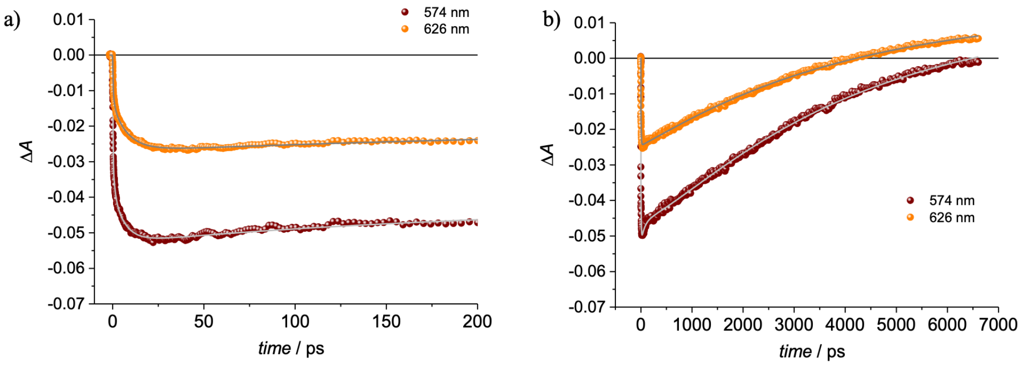

Figure S9: Time evolution of ΔA of C153 in methanol at selected wavelengths on a) short time scale (0-500 ps) and b) long time scale (0-7000 ps). The fittings are reported as lines.

F.2 Time-resolved Emission

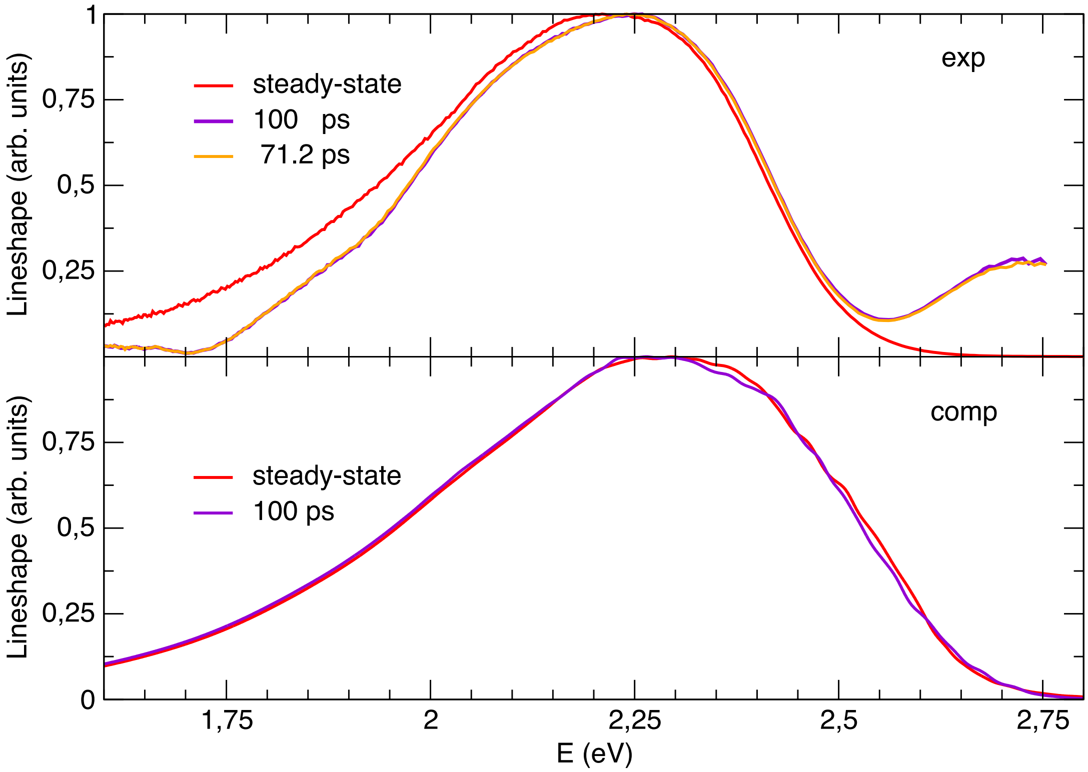

Figure S10: Comparison of steady state emission with the TR one at the longest available time for experimental (top) and MQC computed (bottom) spectra.

F.3 Effect of the temperature of QM modes on TR spectra

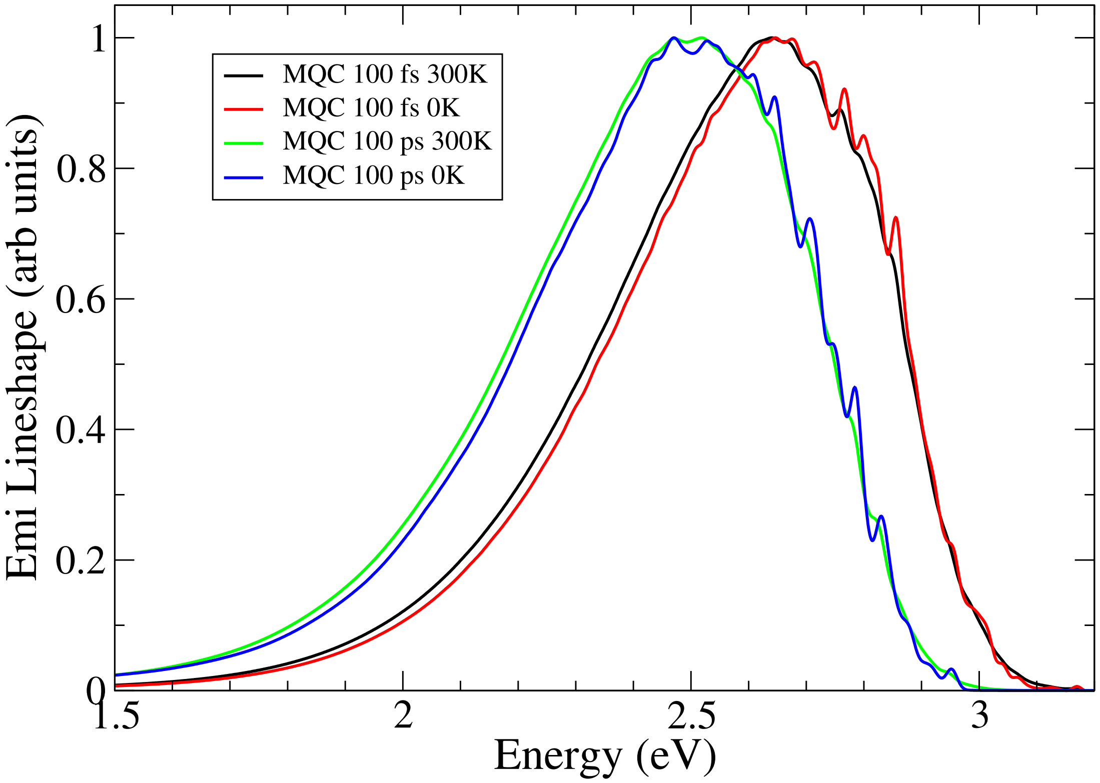

Figure S11: TR emission at short (100 fs) and long (100 ps) times computed asuming a temperature of 0K or 300K for the modes tretated at QM level.

F.4 Solvent Response

Pair correlation functions $g_{\alpha\beta}$ between relevant coumarin atoms (α) and the hydroxyl proton of the methanol solvent (β) were computed over different time intervals and averaged over the 500 non equilibrium runs employed for the transient spectra calculations. Concretely, pair correlation functions were obtained for 100 fs, 400 fs, 1 ps, 2, ps, 4ps, 20 ps and 100 ps by averaging along the MD runs in the [50 - 150 fs], [350 - 450 fs], [0.95 - 1.05 ps], [1.95 - 2.05 ps], [3.95 - 4.05 ps], [19.95 - 20.05 ps] and [99.5- 100.5 ps], respectively.

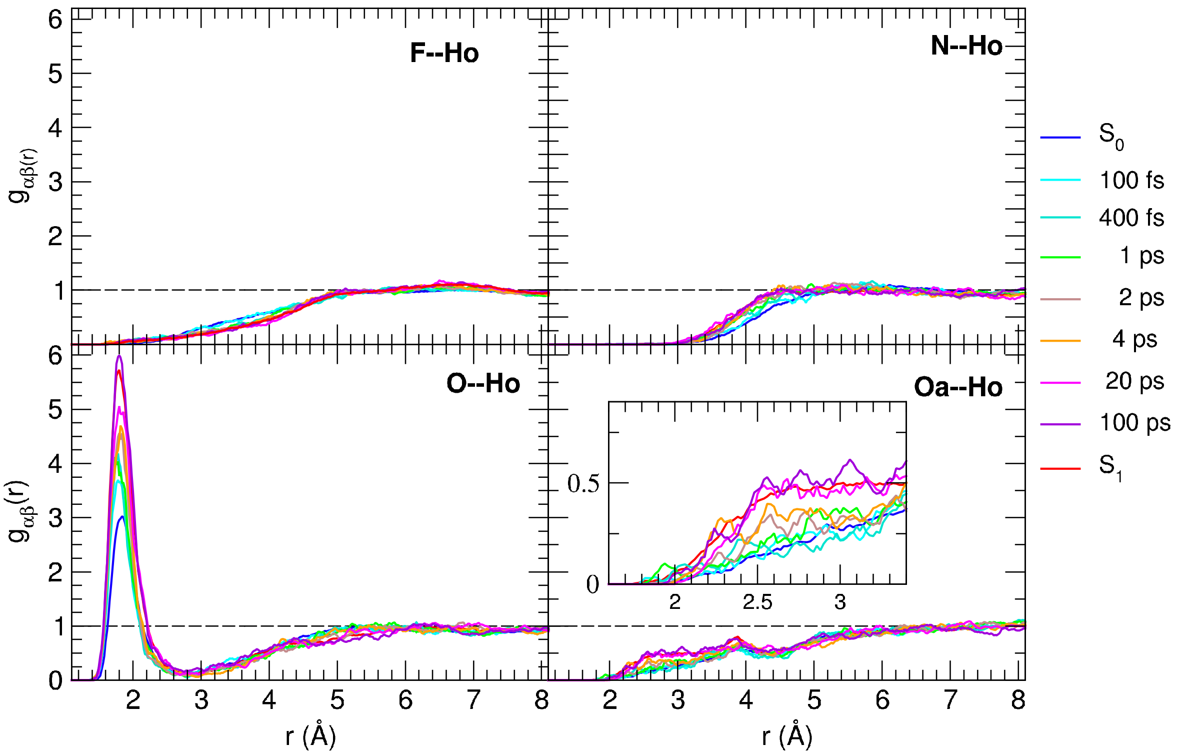

Figure S12: Pair correlation functions between the α C153's atom and the hydroxyl proton (Ho) of the solvent, computed at different time intervals.

References

[1] Jorgensen, W. L.; Maxwell, D. S.; Tirado-rives, J. Development and Testing of the OPLS All-Atom Force Field on Conformational Energetics and Properties of Organic Liquids *J. Am. Chem. Soc.* **1996**, *7863*, 11225–11236.

[2] Jorgensen, W. L.; Tirado-Rives, J. Potential Energy Functions for Atomic-Level Simulations of Water and Organic and Biomolecular Systems. *Proc. Natl. Acad. Sci. USA* **2005**, *102*, 6665–70.

[3] Marenich, A. V.; Jerome, S. V.; Cramer, C. J.; Truhlar, D. G. Charge Model 5: An Extension of Hirshfeld Population Analysis for the Accurate Description of Molecular Interactions in Gaseous and Condensed Phases *J. Chem. Theory Comput.* **2012**, *8*, 527–541.

[4] Tomasi, J.; Mennucci, B.; Cammi, R. Quantum Mechanical Continuum Solvation Models *Chemical Reviews* **2005**, *105*, 2999–3094.

[5] Cacelli, I.; Prampolini, G. Parametrization and Validation of Intramolecular Force Fields Derived from DFT Calculations *J. Chem. Theory Comput.* **2007**, *3*, 1803–1817.

[6] Barone, V.; Cacelli, I.; De Mitri, N.; Licari, D.; Monti, S.; Prampolini, G. Joyce and Ulysses: Integrated and User-Friendly Tools for the Parameterization of Intramolecular Force Fields from Quantum Mechanical Data. *Phys. Chem. Chem. Phys.* **2013**, *15*, 3736–51.

[7] Cerezo, J.; Prampolini, G.; Cacelli, I. Developing accurate intramolecular force fields for conjugated systems through explicit coupling terms *Theor. Chem. Accounts* **2018**, *137*, 80.

[8] Cerezo, J.; Aranda, D.; Avila Ferrer, F. J.; Prampolini, G.; Santoro, F. Adiabatic-Molecular Dynamics Generalized Vertical Hessian Approach: A Mixed Quantum Classical Method to Compute Electronic Spectra of Flexible Molecules in the Condensed Phase *J. Chem. Theory Comput.* **2020**, *16*, 1215–1231.

[9] Cacelli, I.; Cerezo, J.; ; De Mitri, N.; Prampolini, G.; JOYCE2.10, a Fortran 77 code for intra-molecular force field parameterization. , available free of charge at http://www.pi.iccom.cnr.it/joyce, last consulted July; 2020.

[10] Bussi, G.; Donadio, D.; Parrinello, M. Canonical sampling through velocity rescaling *J. Chem. Phys.* **2007**, *126*, 014101.

[11] Parrinello, M.; Rahman, A. Polymorphic transitions in single crystals: A new molecular dynamics method *Journal of Applied Physics* **1981**, *52*, 7182–7190.

[12] M´uhlpfordt, A.; Schanz, R.; P. Ernsting, N.; Farztdinov, V.; Grimme, S. Coumarin 153 in the gas phase: optical spectra and quantum chemical calculations *Phys. Chem. Chem. Phys.* **1999**, *1*, 3209–3218.

[13] Avila Ferrer, F. J.; Cerezo, J.; Soto, J.; Improta, R.; Santoro, F. First-principle computation of absorption and fluorescence spectra in solution accounting for vibronic structure, temperature effects and solvent inhomogenous broadening *Comput. Theoret. Chem.* **2014**, *1040–1041*, 328–337.

[14] Lax, M. The Franck-Condon Principle and Its Application to Crystals *J. Chem. Phys.* **1952**, *20*, 1752–1760.
